# Supplementary material for: Dimerization of GAS2 mediates crosslinking of microtubules and F-actin
Source: EMBO J. 2025 Apr 1;44(10):2997–3024. doi: 10.1038/s44318-025-00415-2 (PMC12084551; doi:10.1038/s44318-025-00415-2)
Supplement: Supplementary file 3 — Appendix [file 44318_2025_415_MOESM3_ESM.pdf]

Appendix

Dimerization of GAS2 mediates crosslinking of microtubules and F-actin

Contents:

Appendix Figure S1

Appendix Figure S2

Appendix Figure S3

Appendix Figure S4

Appendix Figure S5

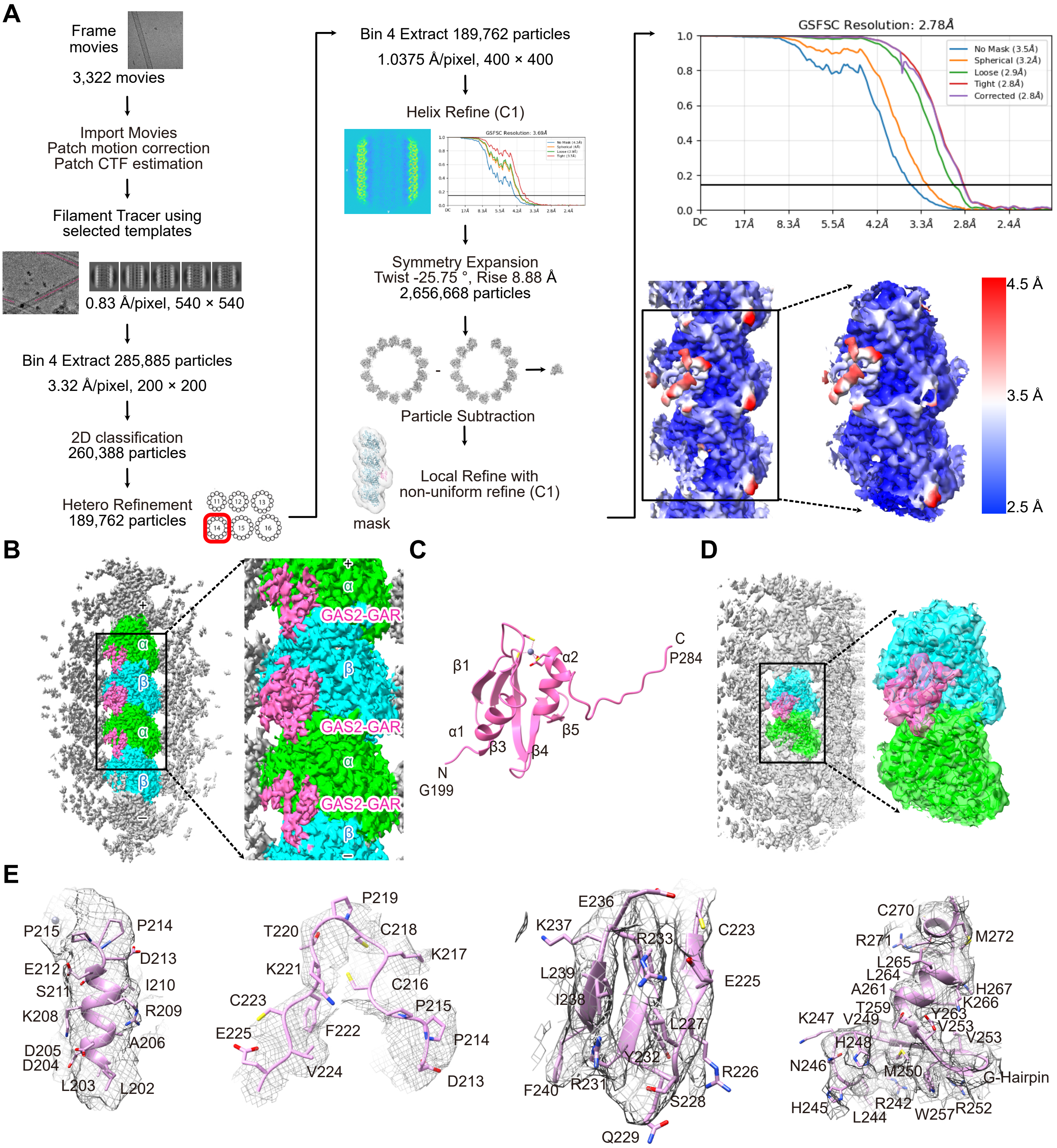

Appendix Figure S1 (**A**) GAS2-GAR-MT structure rebuilding workflow by CryoSPARC. (**B**) CryoSPARC reconstructed Cryo-EM map of 3 tubulin dimer decorated with GAS2-GAR domain. GAS2-GAR density appeared within both intra- and inter-tubulin dimer regions. α-tubulin: lime, β-tubulin: cyan, and GAS2-GAR domain: hot-pink. (**C**) One represents NMR structure of GAS2-GAR domain (PDB: 1V5R, residues A199 to P284). (**D**) Outside top view of atomic models for GAS2-GAR-MT are fitted in CryoSPARC method reconstructed 3D density map (α-tubulin in green, β-tubulin in cyan, GAS2-GAR domain in hot-pink). (**E**) Model-map overlaps for GAS2-GAR-MT. The CryoSPARC method reconstructed GAS2-GAR-MT density map was used, including L202-P215, D213-E225, C223-F240, and R242-M272 regions. Most of the sidechains are fitted well in the CryoSPARC method reconstructed GAS2-GAR-MT density map besides some loops.

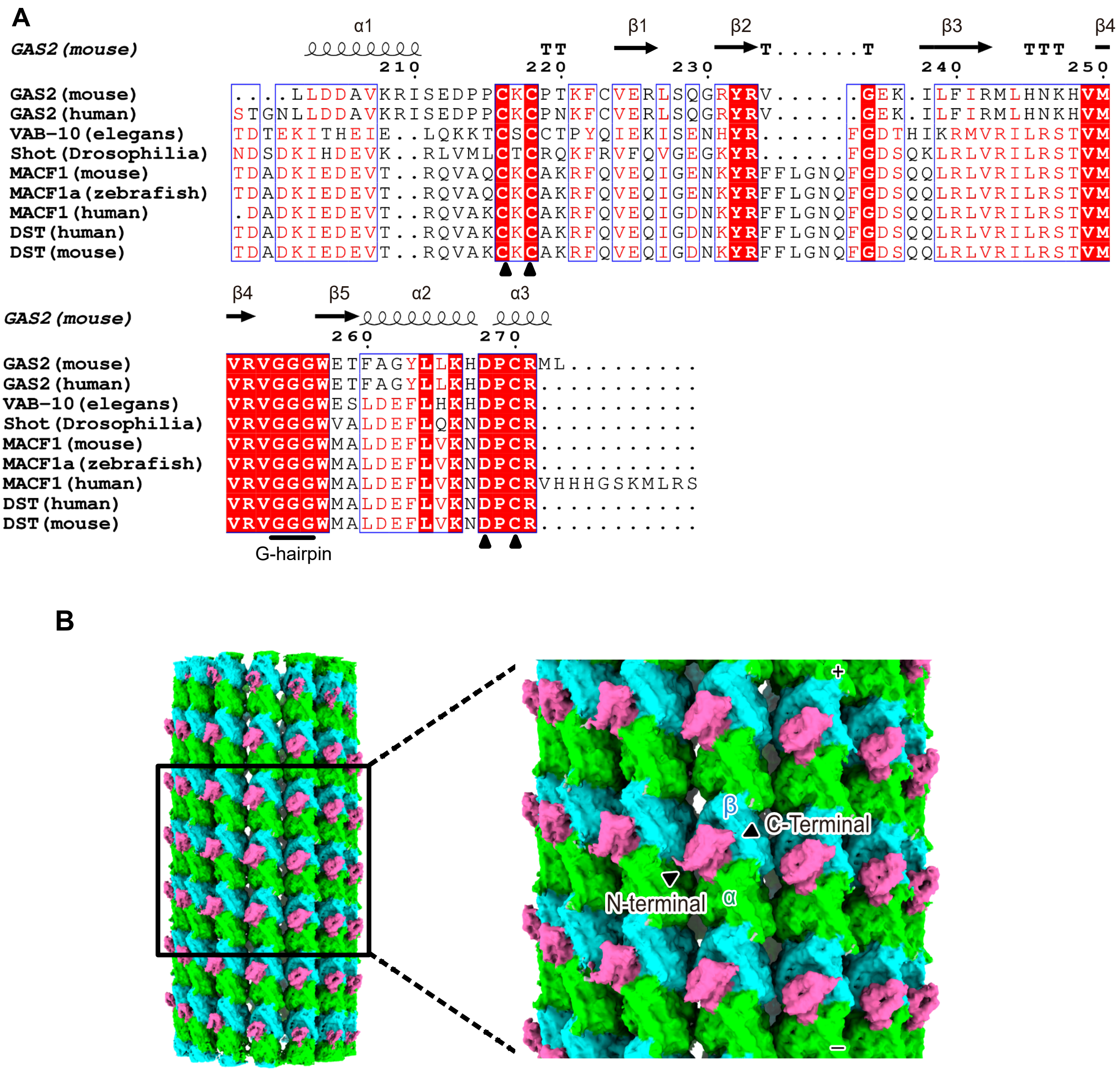

Appendix Figure S2 (**A**) Sequence alignment of the GAR domain from human, mouse, Drosophila melanogaster, *C. elegans*, and zebrafish. The black line shows the conserved G-hairpin. The black triangle indicates the conserved zinc-binding motif. GAS2 (mouse): P11862, GAS2 (human): O43903, VAB-10 (elegans): G5EFM3, Shot (drosophila): Q7KJN8, MACF1 (mouse): Q9QXZ0, MACF1a (zebrafish): A0A8M3AL24, MACF1 (human): Q9UPN3, DST (human): Q03001, DST (mouse): Q91ZU6. (**B**) 4.4 Å cryo-EM structure of one microtubule decorated with GAS2-FL.

A

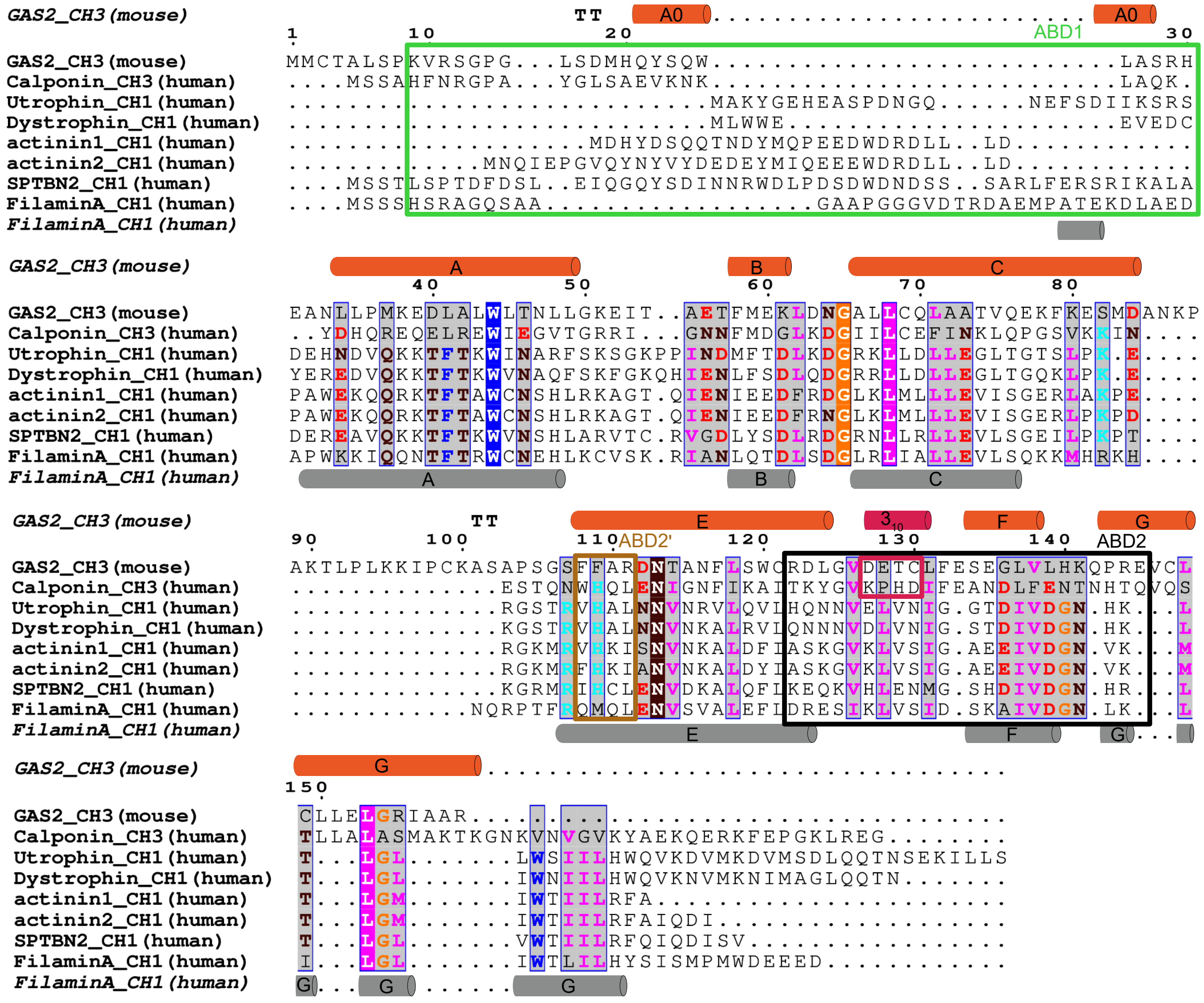

Appendix Figure S3 (**A**) CH1 and CH3 domain sequence alignment. Primary accession on Uniprot for each sequence: GAS2-CH3 (mouse): P11862; Dystrophin (human): P11532; Calponin-1 (human): P51911; Utrophin (human): P46939; ACTN1 (human): P12814; ACTN2 (human): P35609; SPTBN2 (human): O15020; FilaminA (human): P21333. (F) CH3 domain sequence alignment. Primary accession on Uniprot for each sequence: GAS2CH (mouse): P11862; mal3 (yeast): Q10113; EB1 (mouse): Q61166; EB2 (mouse): Q8R001; EB3(mouse): Q6PER3; Calponin-1(human): P51911; Tagln2 (mouse) Q9WVA4. ABD1 sequences were shown in the green box. ABD2' sequences were shown in the brown box. 3_{10} α-helix was shown in the red box. ABD2 sequences were shown in the black box.

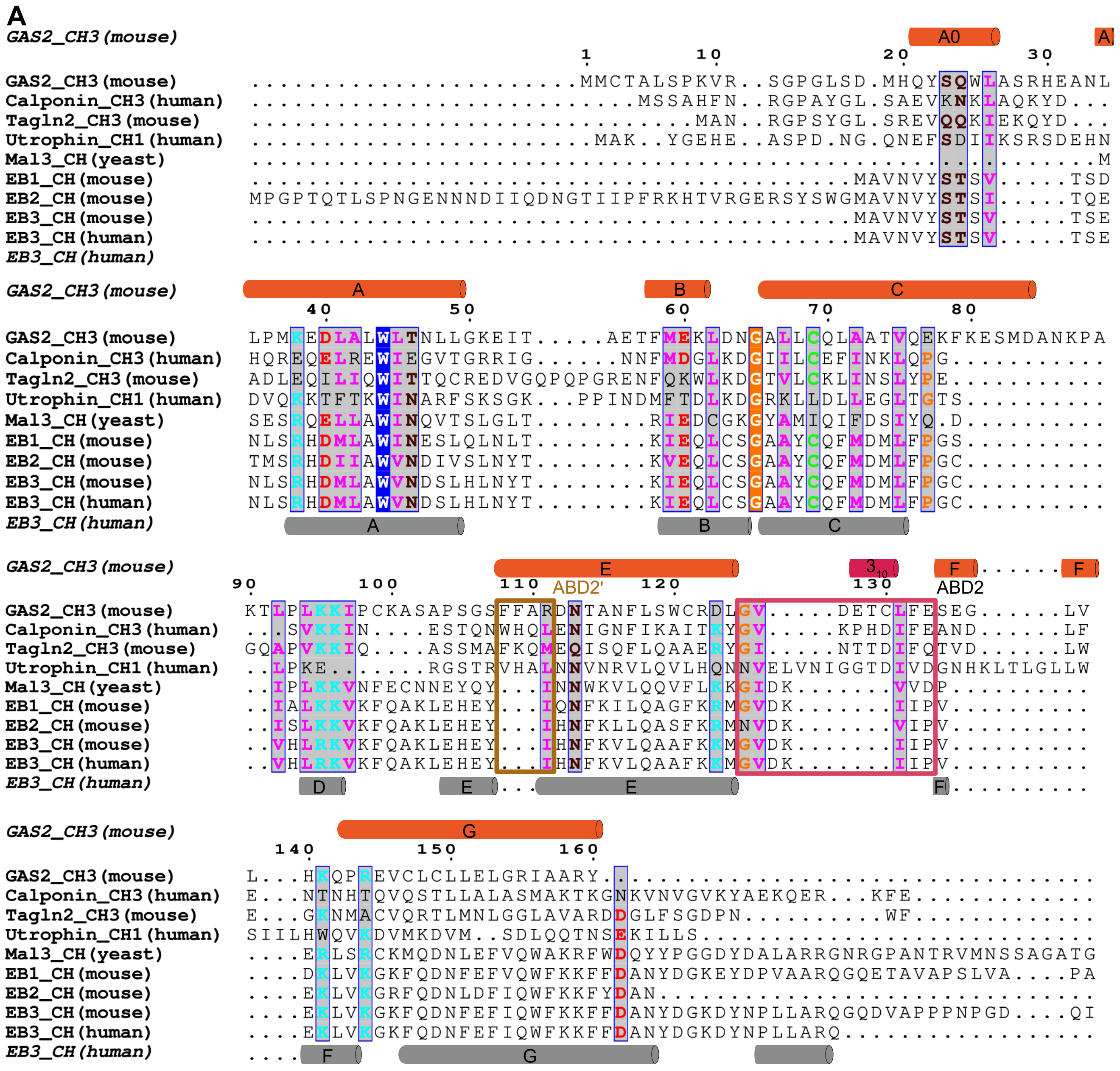

Appendix Figure S4 (**A**) F-actin binding single CH3 domain and MT binding sequence alignment. Primary accession on Uniprot for each sequence: GAS2-CH3 (mouse): P11862; Calponin-1 (human): P51911; Tagln2 (mouse): Q9WVA4, Mal3 (yeast): Q10113; EB1 (mouse): Q61166; EB2 (mouse): Q8R001; EB3 (mouse): Q6PER3; EB3 (human): Q9UPY8. ABD2' sequences were shown in the brown box. Loop sequences between E and F helix were shown in the red box.

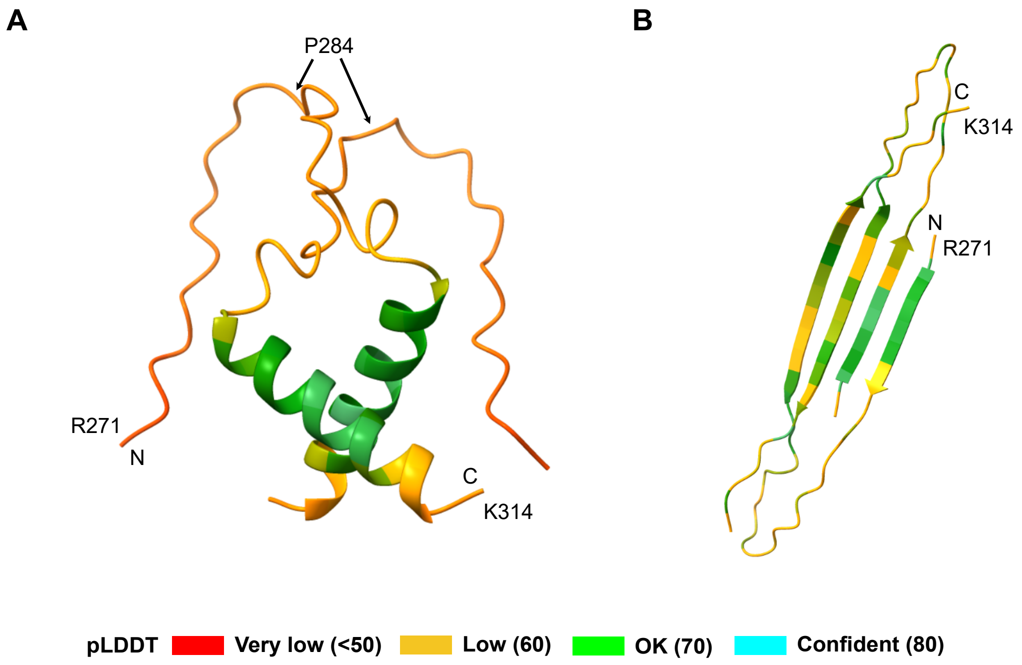

Appendix Figure S5 (**A**) AlphaFold2-predicted dimerization structure of the C-terminal region (R271–K314), color-coded by pLDDT score using a rainbow gradient from the N- to C-terminus. The predicted structure indicates that the R271–P284 segment comprises flexible loops, while the P284–K314 region constitutes the dimerization interface. (**B**) AlphaFold3-predicted dimerization structure of the C-terminal region (R271–K314), similarly color-coded by pLDDT score. The prediction suggests that the C-terminal region adopts a β-sheet hairpin conformation. (Red: Very low, pLDDT score < 50; Orange: Low, pLDDT score > 60; Lime: Ok, pLDDT score > 70; Cyan: Confident, pLDDT score > 80).
