# Supplementary material for: Dimerization of GAS2 mediates crosslinking of microtubules and F-actin
Source: EMBO J. 2025 Apr 1;44(10):2997–3024. doi: 10.1038/s44318-025-00415-2 (PMC12084551; doi:10.1038/s44318-025-00415-2)
Supplement: Supplementary file 5 — Movie EV1 [file 44318_2025_415_MOESM5_ESM.zip › 2024-119009_Movie_EV1/Movie EV1 legend file.docx]

**Movie EV1 Dark-field microscope results of** **2 μM Bare MT.**

**Description:** Dark-field were used to observe bare MT. Taxol-stabilized MTs (2 μM) samples were imaged using the dark-field microscopy (BX53, Olympus). Bare MT is disordered as individual filaments. The scale bar represents 20 μm.
