# Supplementary material for: Dimerization of GAS2 mediates crosslinking of microtubules and F-actin
Source: EMBO J. 2025 Apr 1;44(10):2997–3024. doi: 10.1038/s44318-025-00415-2 (PMC12084551; doi:10.1038/s44318-025-00415-2)
Supplement: Supplementary file 8 — Movie EV4 [file 44318_2025_415_MOESM8_ESM.zip › 2024-119009_Movie_EV4/Movie EV4 legend file.docx]

**Movie EV4 Dark-field microscope results of** **1 μM GAS2ΔC couldn't bundle MT.**

**Description:** Dark-field were used to observe MT bundling induced by GAS2ΔC. Taxol-stabilized MTs (2 μM) were incubated with 1.0 μM GAS2ΔC proteins in BRB80 at room temperature for 30 min. The samples were then imaged using dark-field microscopy (BX53, Olympus). MTs were still disordered as individual filaments. The scale bar represents 20 μm.
