# Supplementary material for: Dimerization of GAS2 mediates crosslinking of microtubules and F-actin
Source: EMBO J. 2025 Apr 1;44(10):2997–3024. doi: 10.1038/s44318-025-00415-2 (PMC12084551; doi:10.1038/s44318-025-00415-2)
Supplement: Supplementary file 10 — Movie EV6 [file 44318_2025_415_MOESM10_ESM.zip › 2024-119009_Movie_EV6/Movie EV6 legend file.docx]

**Movie EV6**

**Representative videos of TIRF assays for tubulin alone.**

**Description:** The assay features 10 µM rhodamine-labeled tubulin (magenta) polymerized in the presence of randomly oriented short GMPCPP-stabilized MT seeds (not shown in the video). The scale bar represents 5 µm. The total imaging duration is 20 minutes. The video is representative of 7 independent experiments.
