# Supplementary material for: Dimerization of GAS2 mediates crosslinking of microtubules and F-actin
Source: EMBO J. 2025 Apr 1;44(10):2997–3024. doi: 10.1038/s44318-025-00415-2 (PMC12084551; doi:10.1038/s44318-025-00415-2)
Supplement: Supplementary file 11 — Movie EV7 [file 44318_2025_415_MOESM11_ESM.zip › 2024-119009_Movie_EV7/Movie EV7 legend file.docx]

**Movie EV7**

**Representative videos of TIRF-based assays for tubulin in the presence of GAS2-GAR-GFP.**
**Description:** The assay demonstrates the MTs (magenta, 10 µM) were decorated by GAS2-GAR-GFP (green, 100 nM) in the context of randomly oriented short GMPCPP-stabilized MT seeds (not shown in the video). The scale bar represents 5 µm. The total imaging duration is 20 minutes. The video is representative of 12 independent experiments.
