# Supplementary material for: Dimerization of GAS2 mediates crosslinking of microtubules and F-actin
Source: EMBO J. 2025 Apr 1;44(10):2997–3024. doi: 10.1038/s44318-025-00415-2 (PMC12084551; doi:10.1038/s44318-025-00415-2)
Supplement: Supplementary file 13 — Movie EV9 [file 44318_2025_415_MOESM13_ESM.zip › 2024-119009_Movie_EV9/Movie EV9 legend file.docx]

**Movie EV9**

**Representative videos of GAS2-GAR inducing microtubule behavior in vitro. Description:** Microtubule growth from a spot-like aggregation (cyan arrow) in the presence of GAS2-GAR. The yellow arrow represents the MT bundles. The white arrow represents the single MT filament. The scale bar represents 5 µm. The total imaging duration is 3 min. The video is representative of 3 independent experiments.
