# Supplementary figures and images for: Dimerization of GAS2 mediates crosslinking of microtubules and F-actin
Source: EMBO J. 2025 Apr 1;44(10):2997–3024. doi: 10.1038/s44318-025-00415-2 (PMC12084551; doi:10.1038/s44318-025-00415-2)

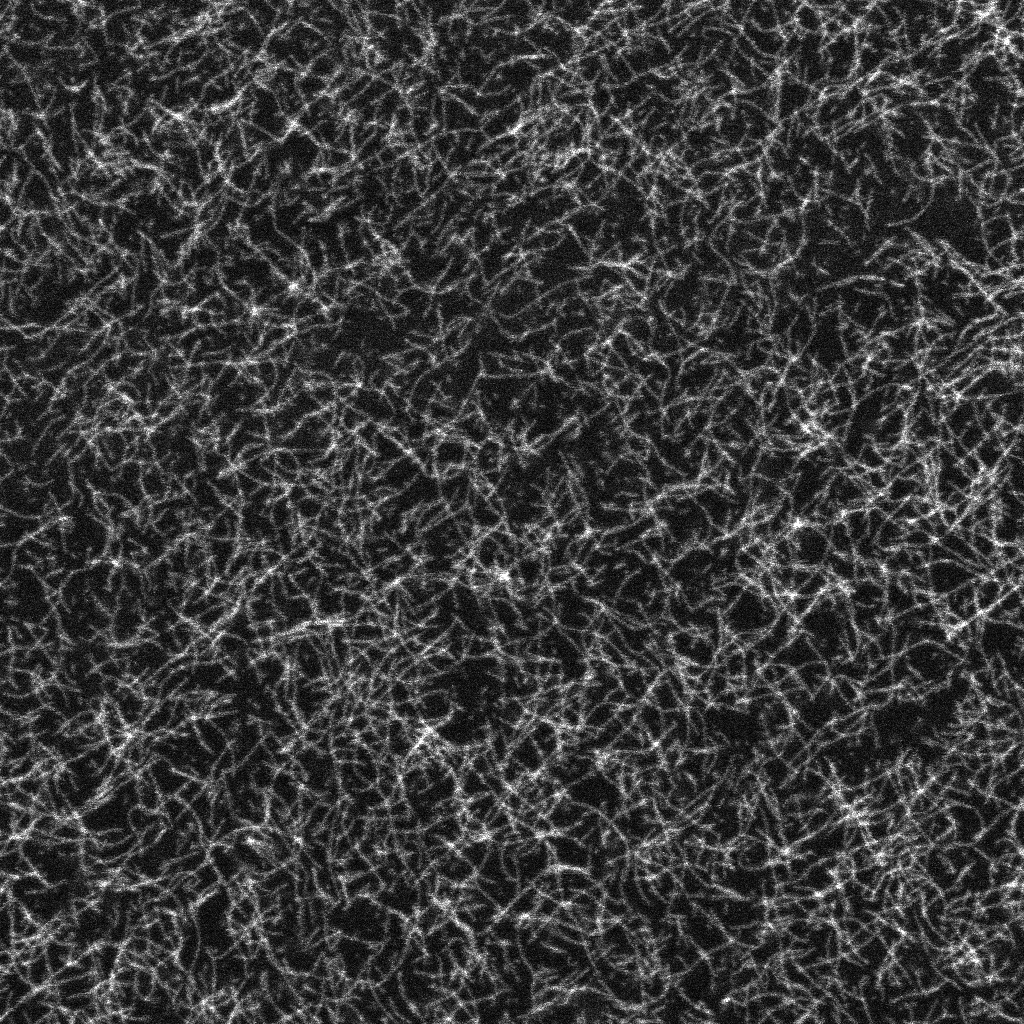

Supplement: Supplementary file 15 — Source data Fig. 1 [file 44318_2025_415_MOESM15_ESM.zip › Figure1/1D/Figure 1D.tif]

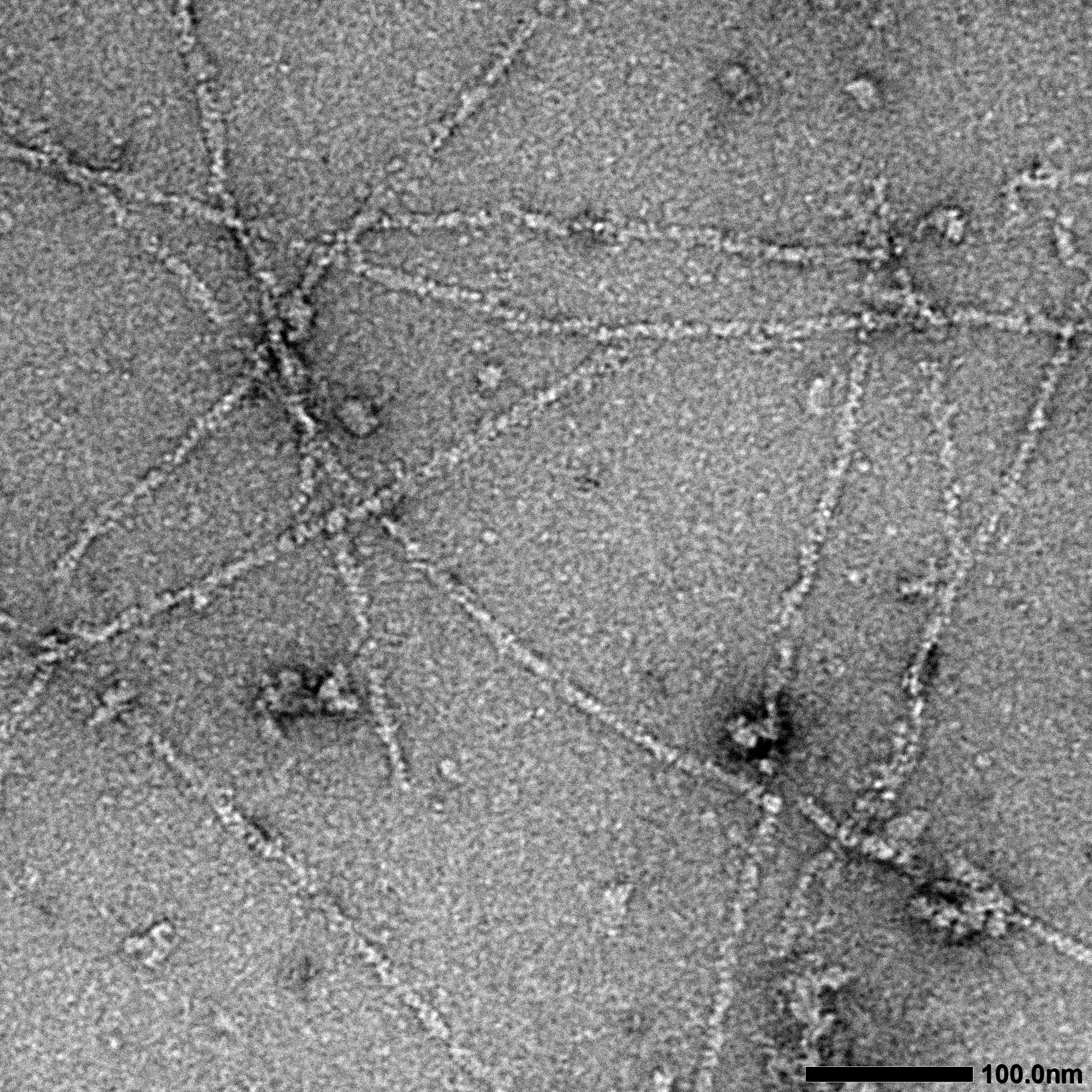

Supplement: Supplementary file 15 — Source data Fig. 1 [file 44318_2025_415_MOESM15_ESM.zip › Figure1/1I/Figure 1I.bmp]

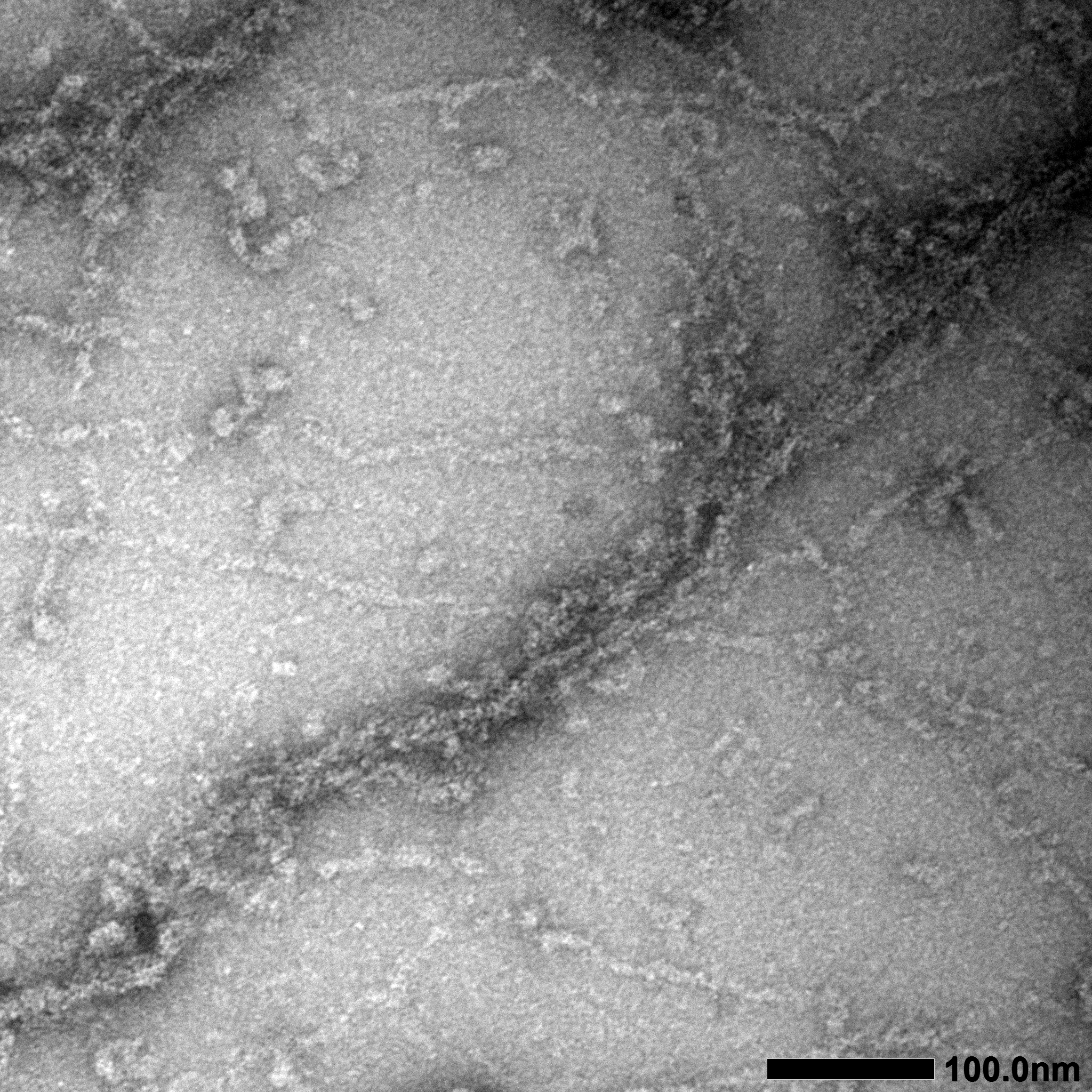

Supplement: Supplementary file 15 — Source data Fig. 1 [file 44318_2025_415_MOESM15_ESM.zip › Figure1/1J/Figure 1J.bmp]

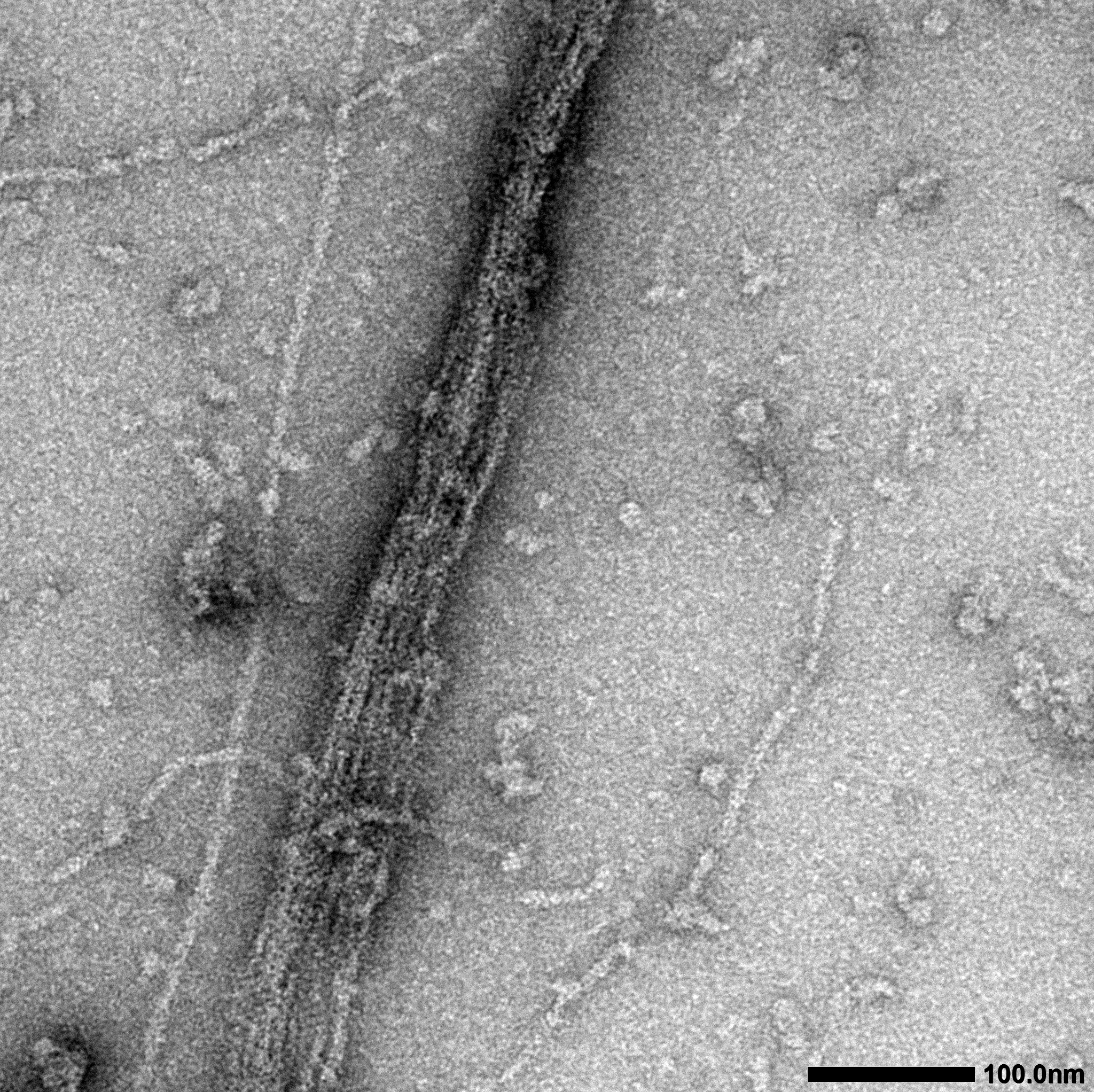

Supplement: Supplementary file 15 — Source data Fig. 1 [file 44318_2025_415_MOESM15_ESM.zip › Figure1/1K/Figure 1K.bmp]

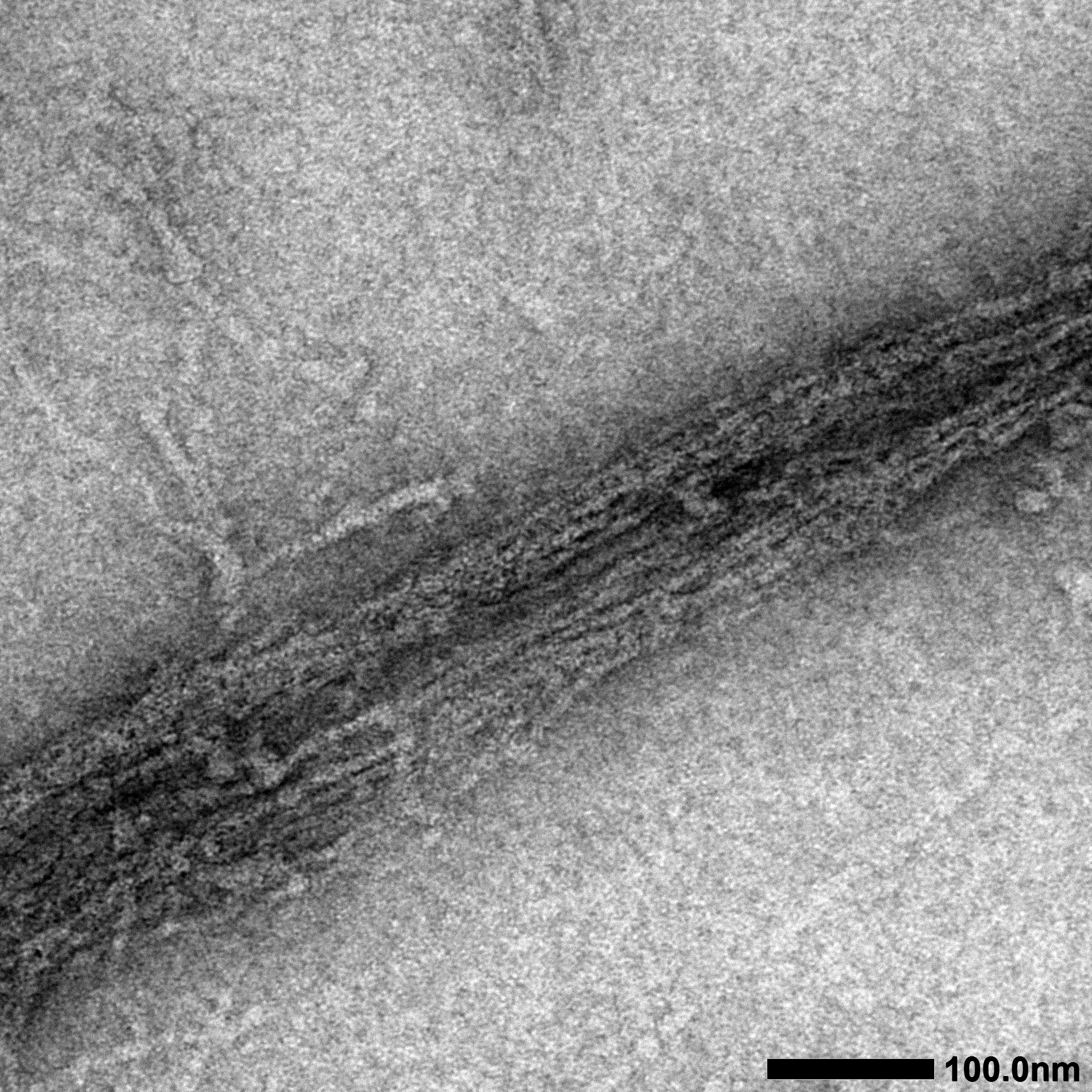

Supplement: Supplementary file 15 — Source data Fig. 1 [file 44318_2025_415_MOESM15_ESM.zip › Figure1/1L/Figure 1L.bmp]

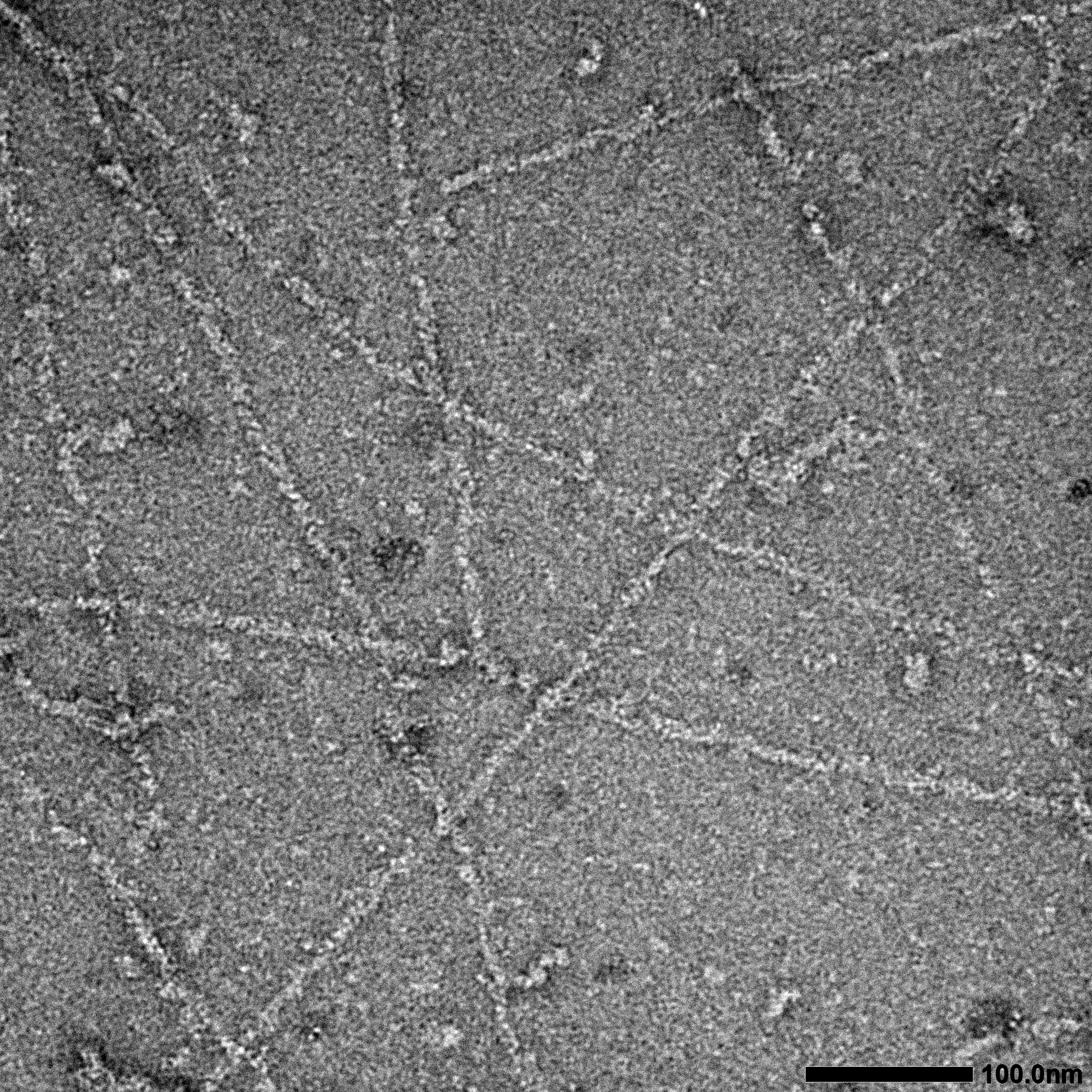

Supplement: Supplementary file 15 — Source data Fig. 1 [file 44318_2025_415_MOESM15_ESM.zip › Figure1/1M/Figure 1M.bmp]

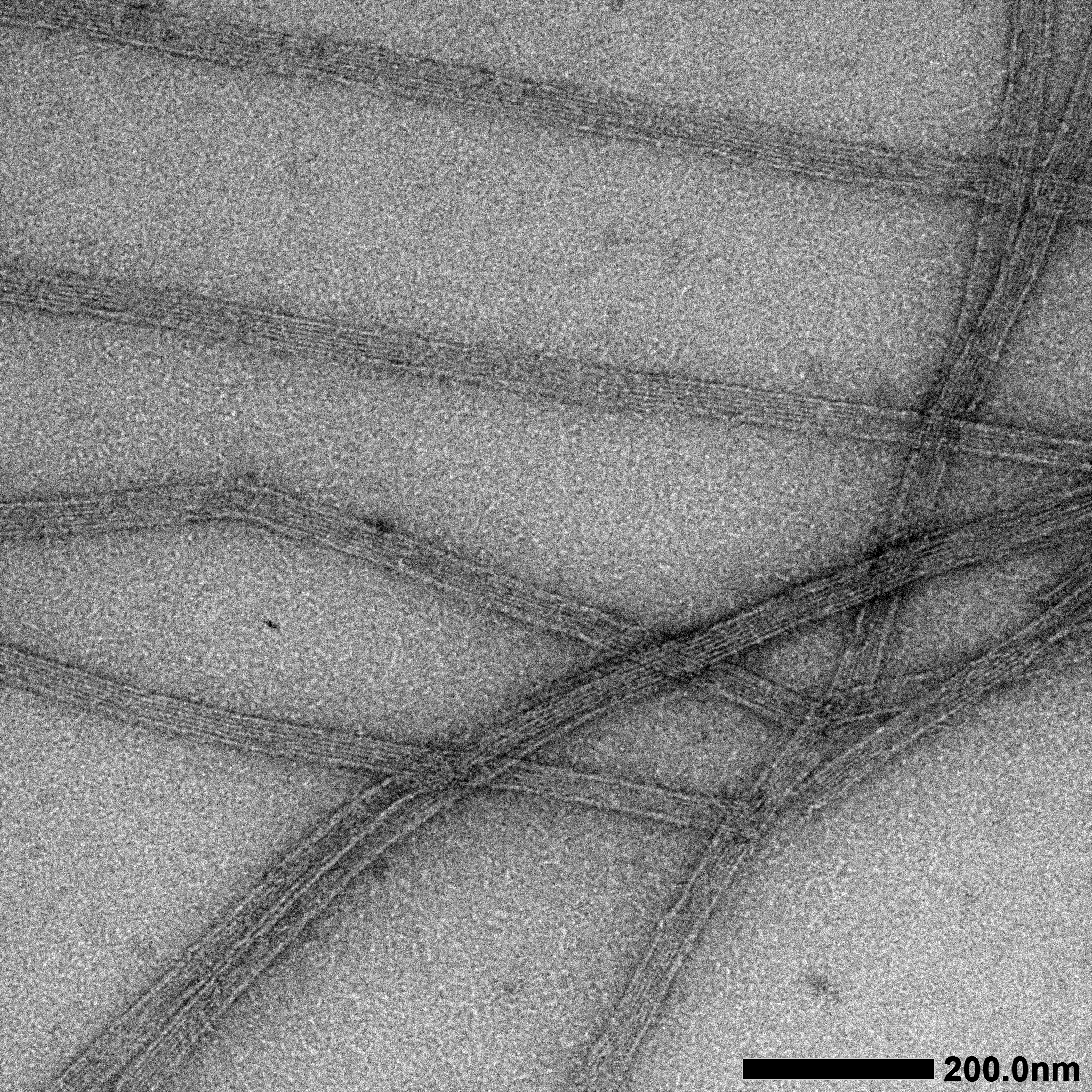

Supplement: Supplementary file 17 — Source data Fig. 3 [file 44318_2025_415_MOESM17_ESM.zip › Figure3/3A/Figure 3A.bmp]

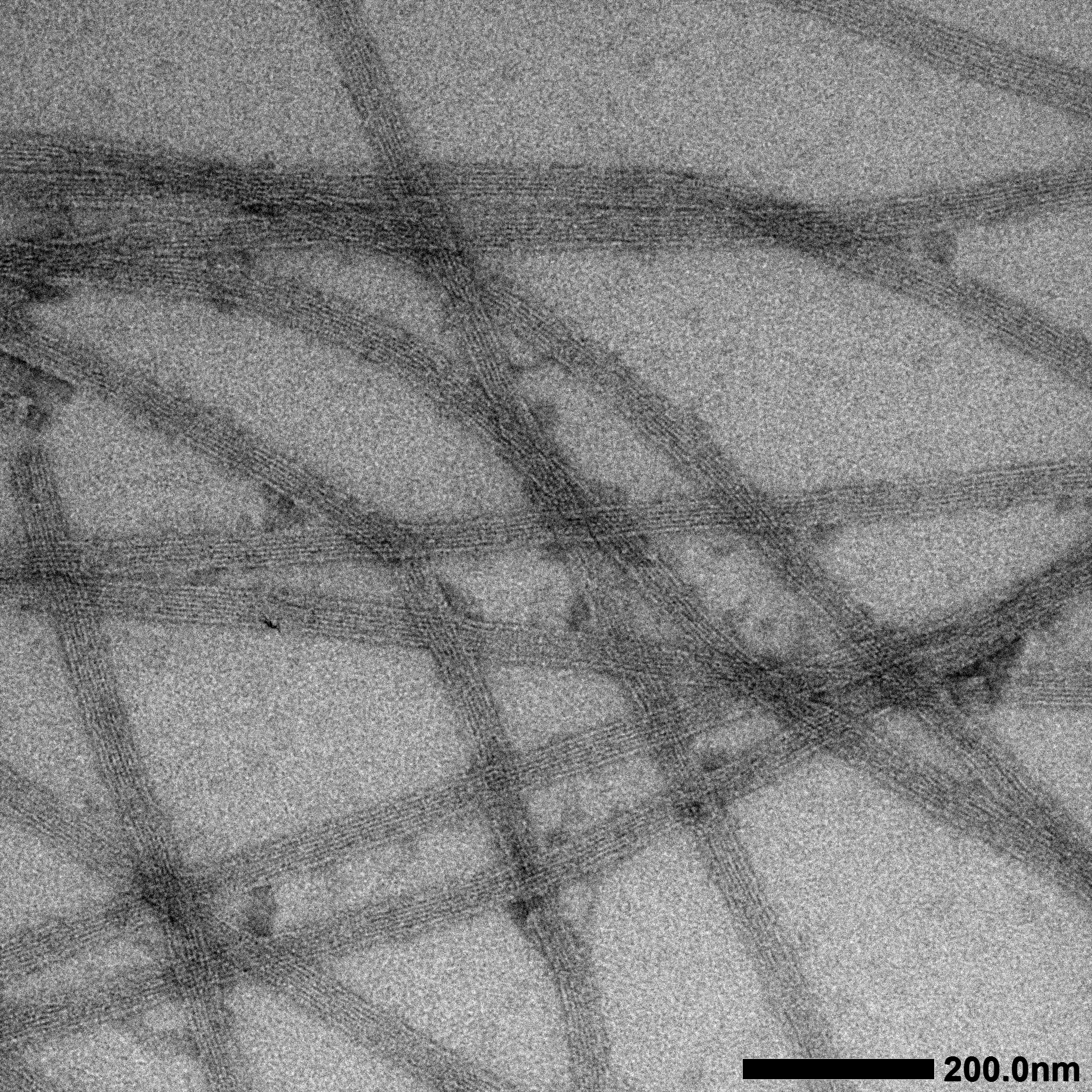

Supplement: Supplementary file 17 — Source data Fig. 3 [file 44318_2025_415_MOESM17_ESM.zip › Figure3/3B/Figure 3B.bmp]

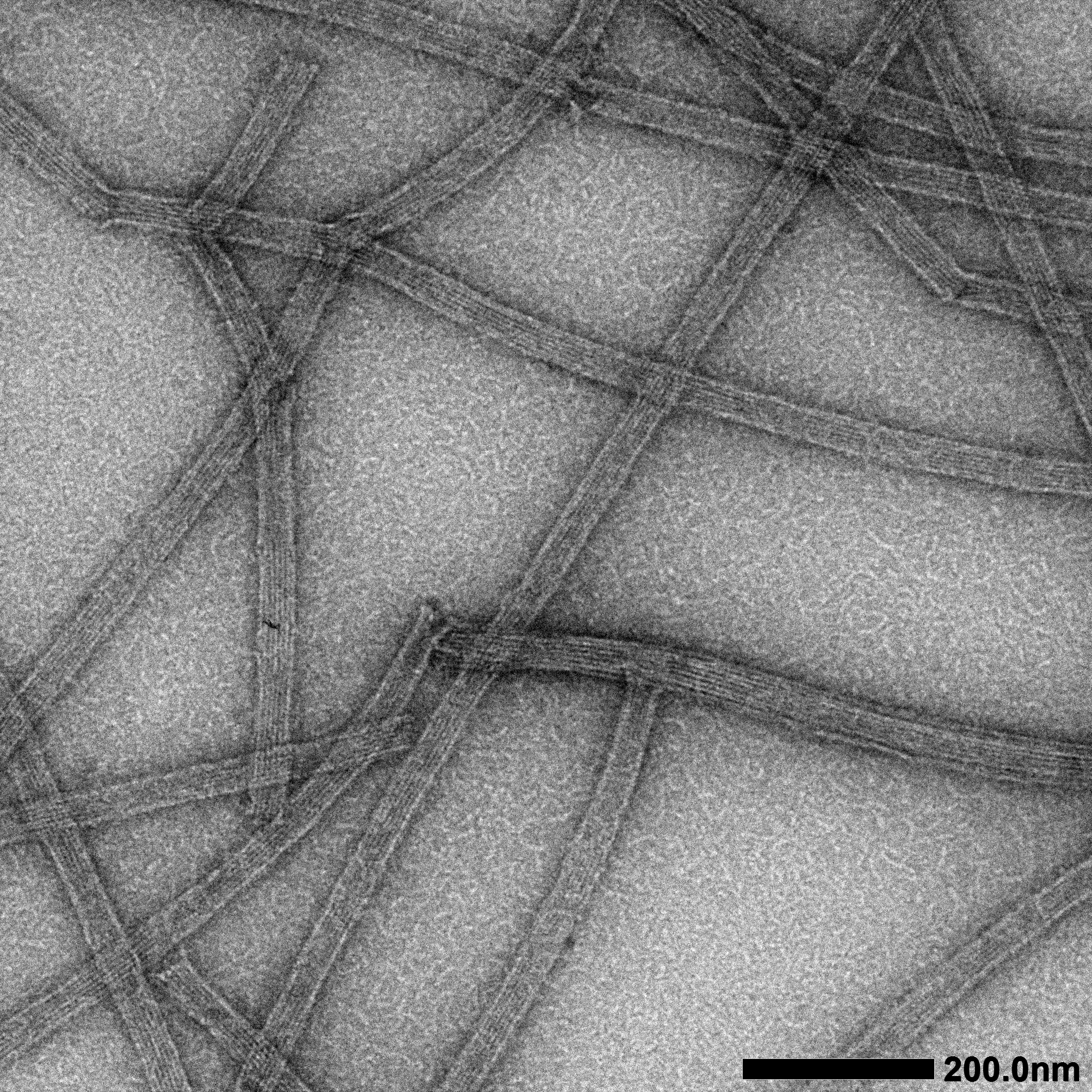

Supplement: Supplementary file 17 — Source data Fig. 3 [file 44318_2025_415_MOESM17_ESM.zip › Figure3/3C/Figure 3C.bmp]

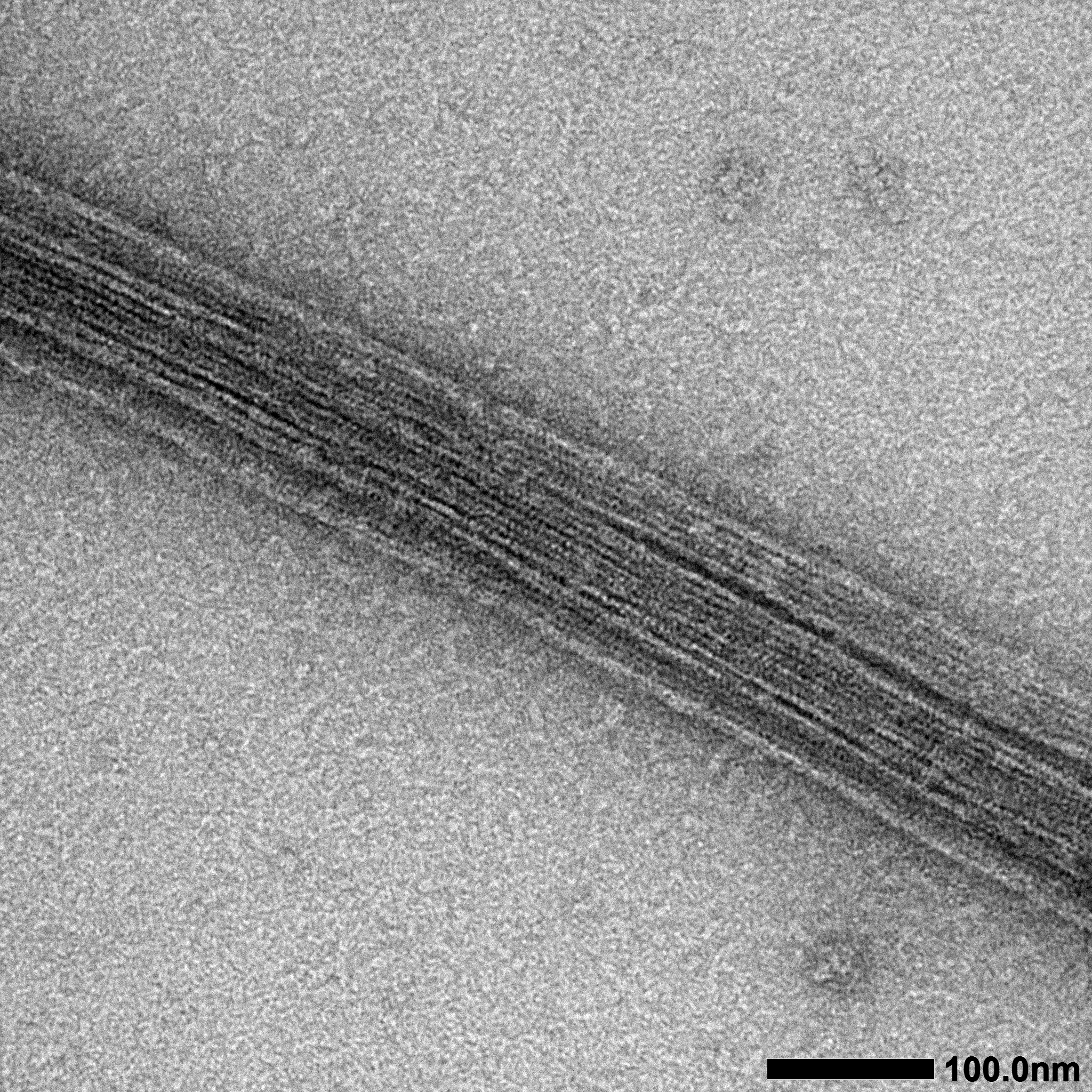

Supplement: Supplementary file 17 — Source data Fig. 3 [file 44318_2025_415_MOESM17_ESM.zip › Figure3/3D/Figure 3D.bmp]

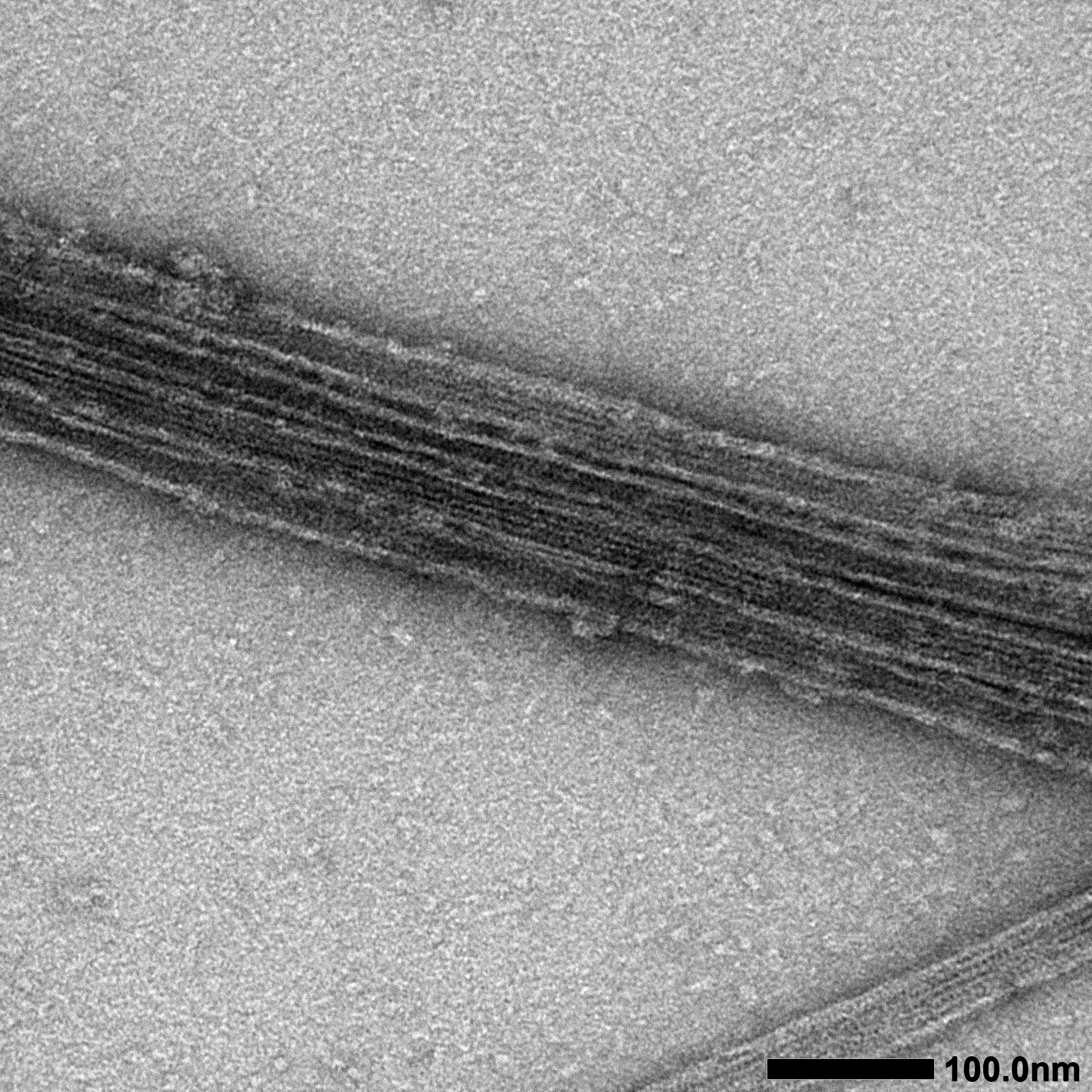

Supplement: Supplementary file 17 — Source data Fig. 3 [file 44318_2025_415_MOESM17_ESM.zip › Figure3/3E/Figure 3E.bmp]

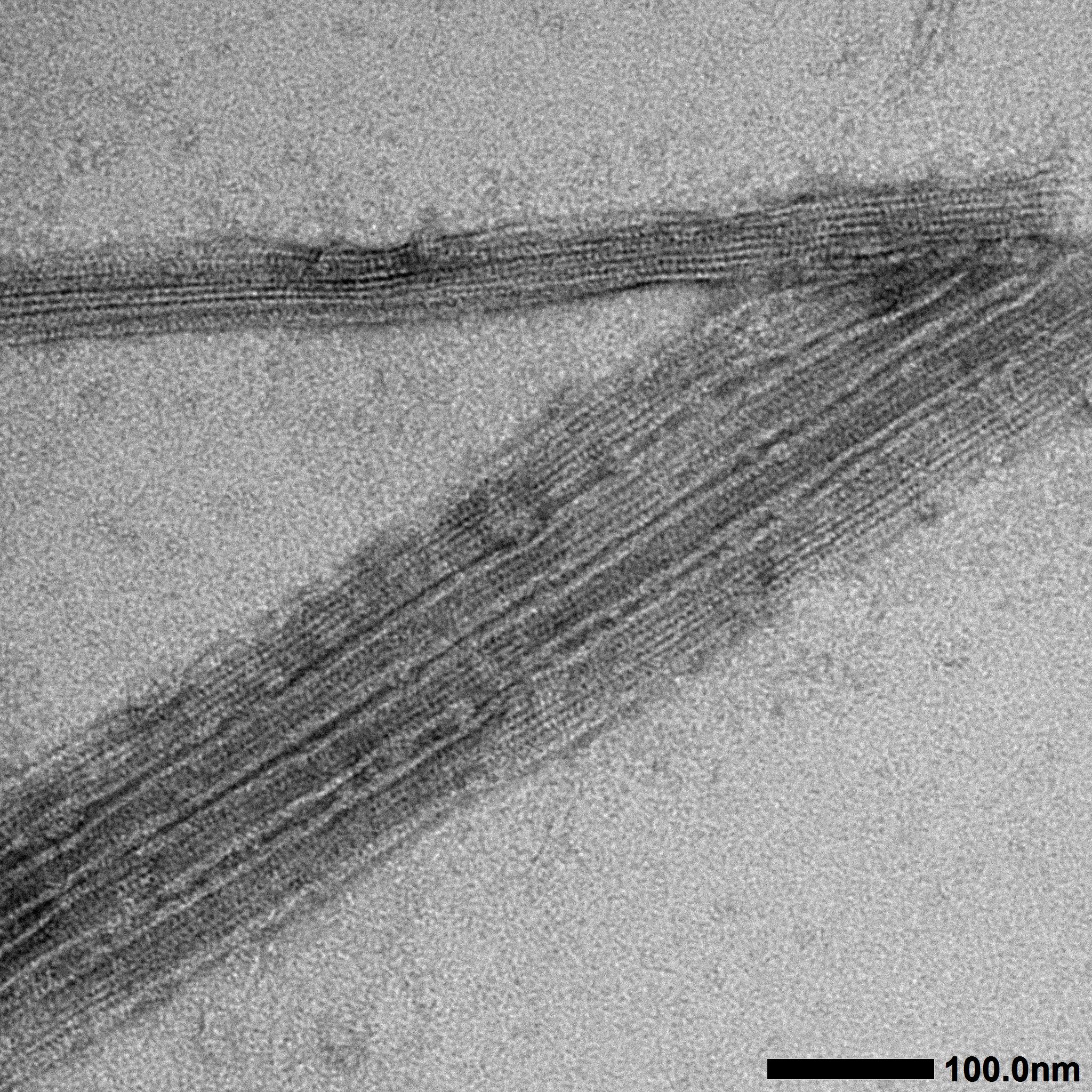

Supplement: Supplementary file 17 — Source data Fig. 3 [file 44318_2025_415_MOESM17_ESM.zip › Figure3/3F/Figure 3F.jpg]

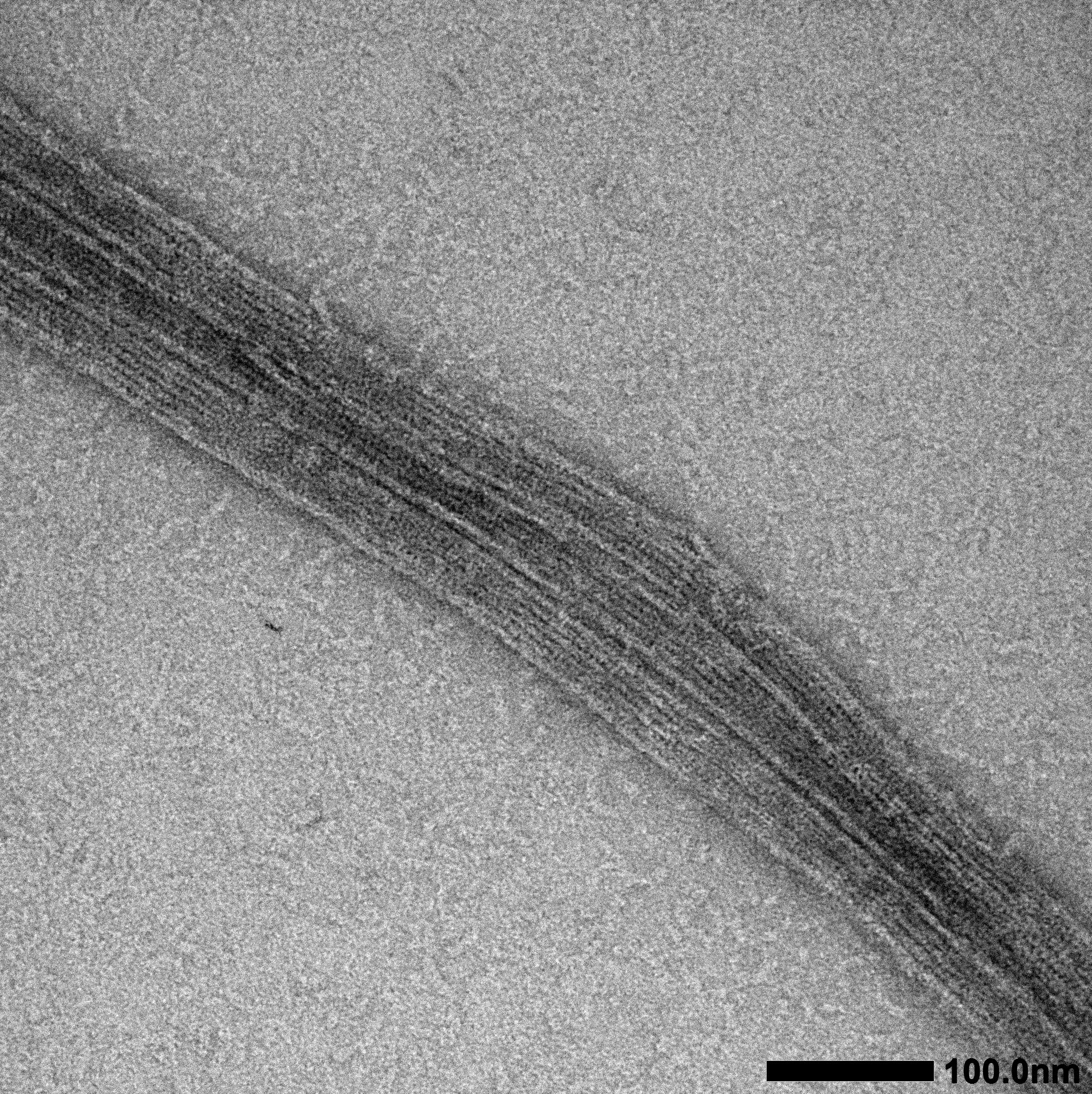

Supplement: Supplementary file 17 — Source data Fig. 3 [file 44318_2025_415_MOESM17_ESM.zip › Figure3/3G/Figure 3G.bmp]

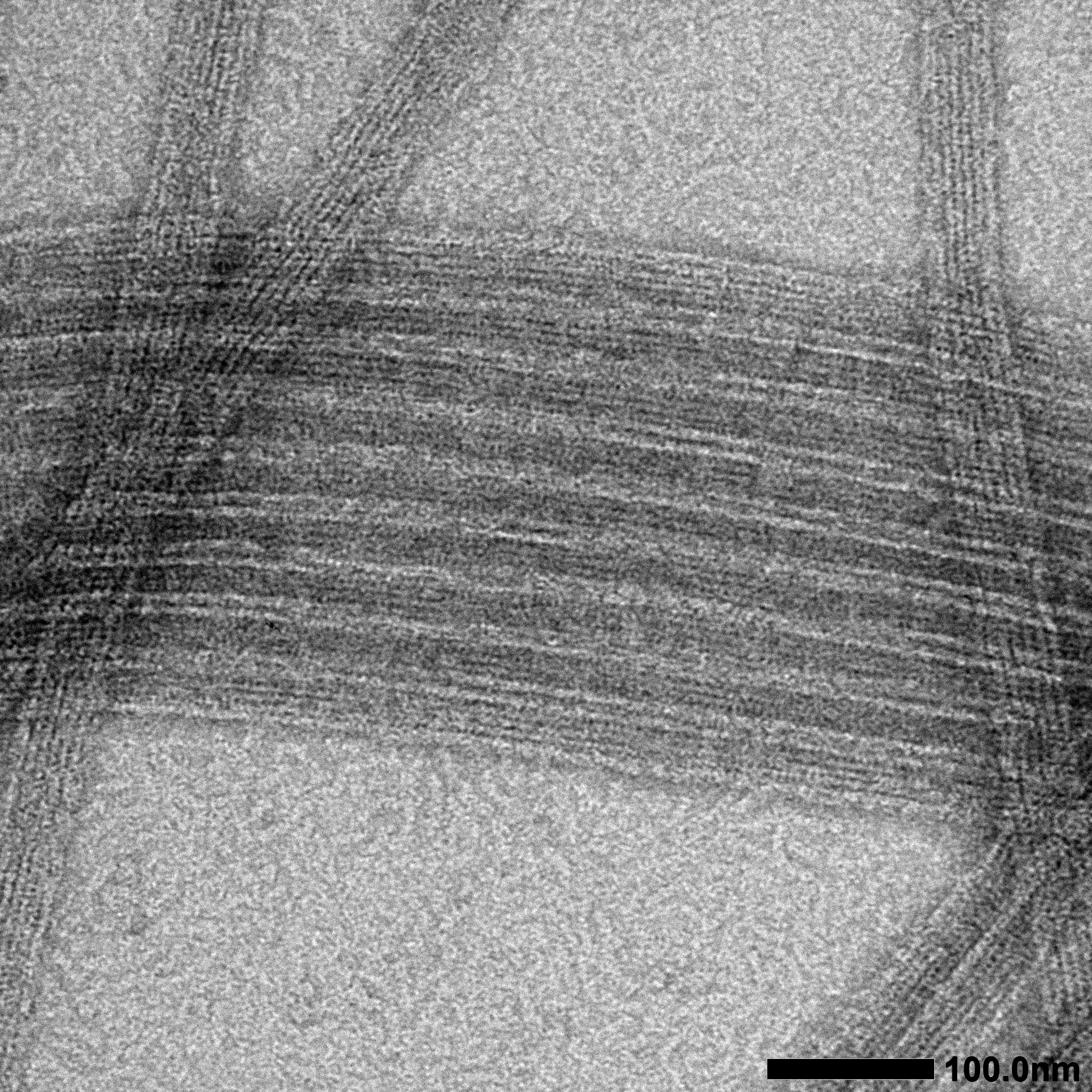

Supplement: Supplementary file 17 — Source data Fig. 3 [file 44318_2025_415_MOESM17_ESM.zip › Figure3/3H/Figure 3H.bmp]

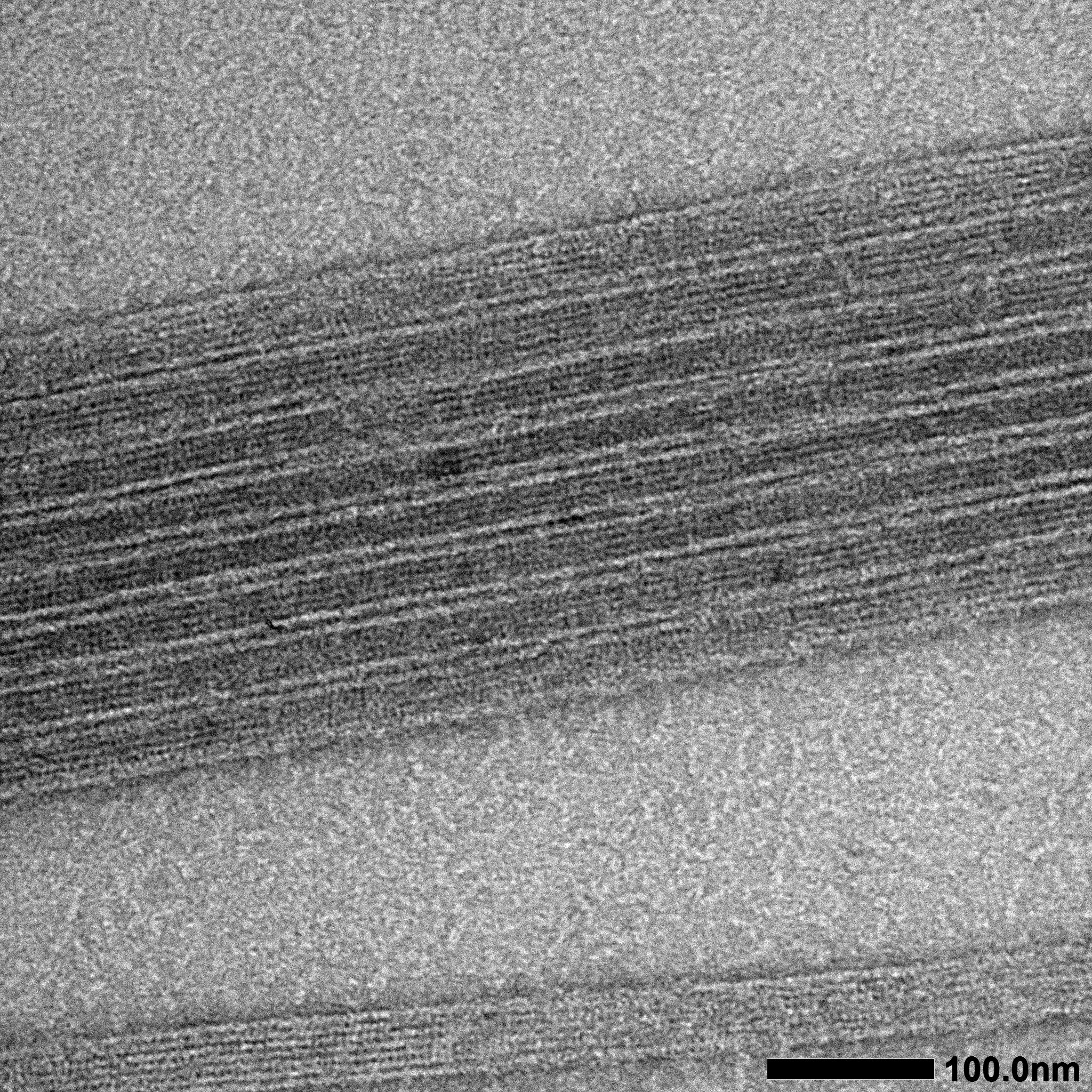

Supplement: Supplementary file 17 — Source data Fig. 3 [file 44318_2025_415_MOESM17_ESM.zip › Figure3/3I/Figure 3I.bmp]

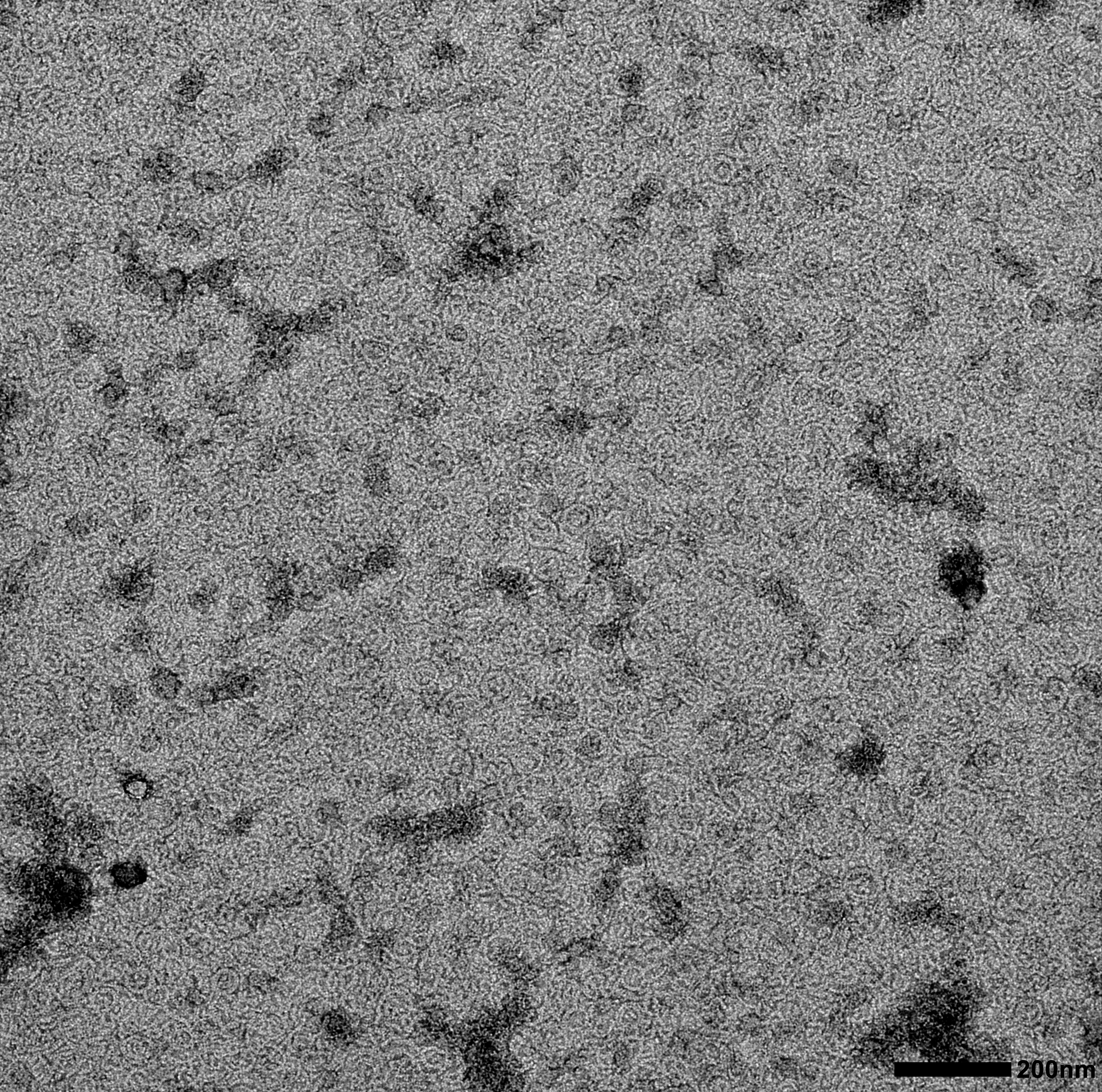

Supplement: Supplementary file 20 — Source data Fig. 6 [file 44318_2025_415_MOESM20_ESM.zip › Figure6/6A/Figure 6A.bmp]

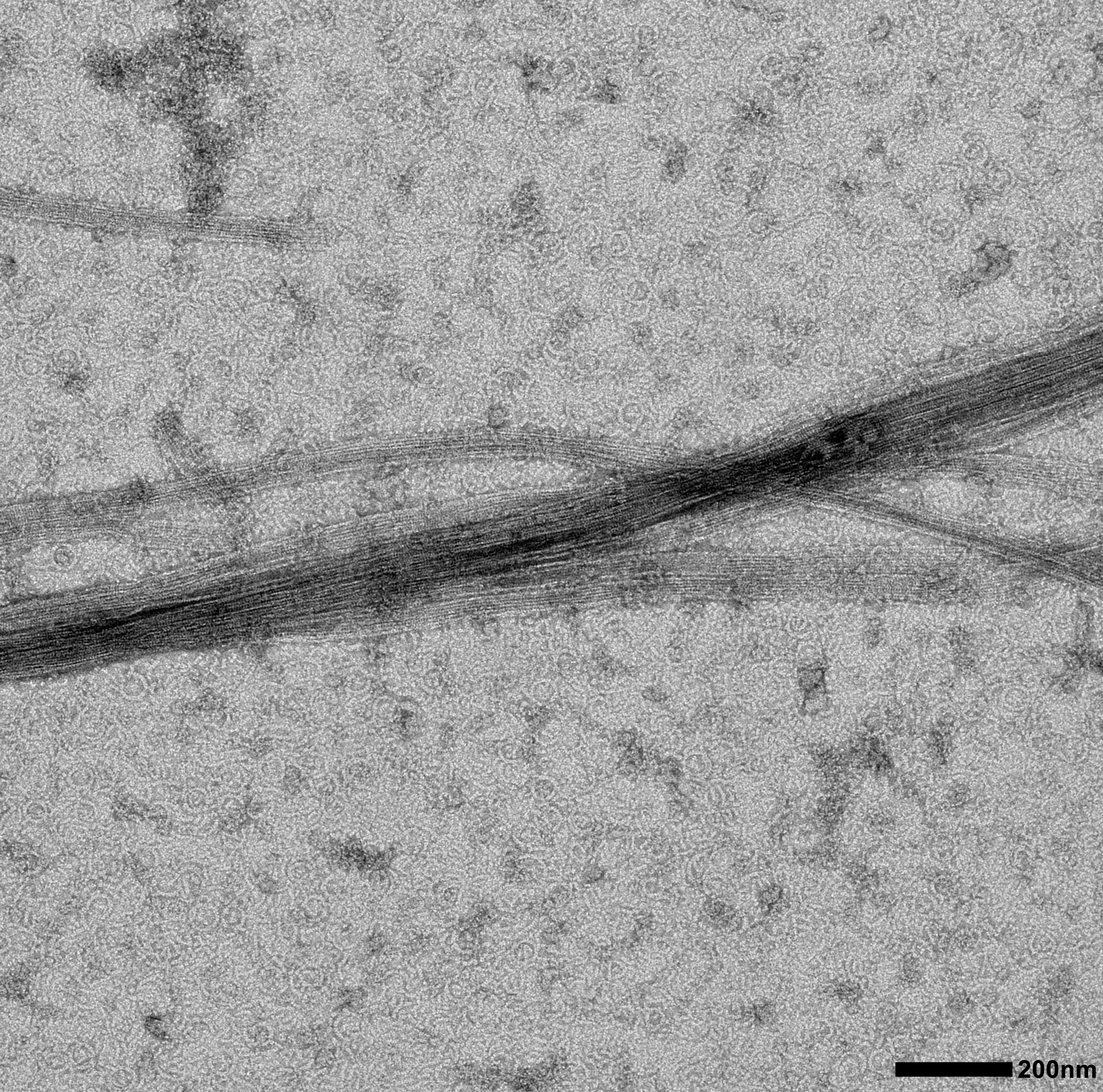

Supplement: Supplementary file 20 — Source data Fig. 6 [file 44318_2025_415_MOESM20_ESM.zip › Figure6/6B/Figure 6B.bmp]

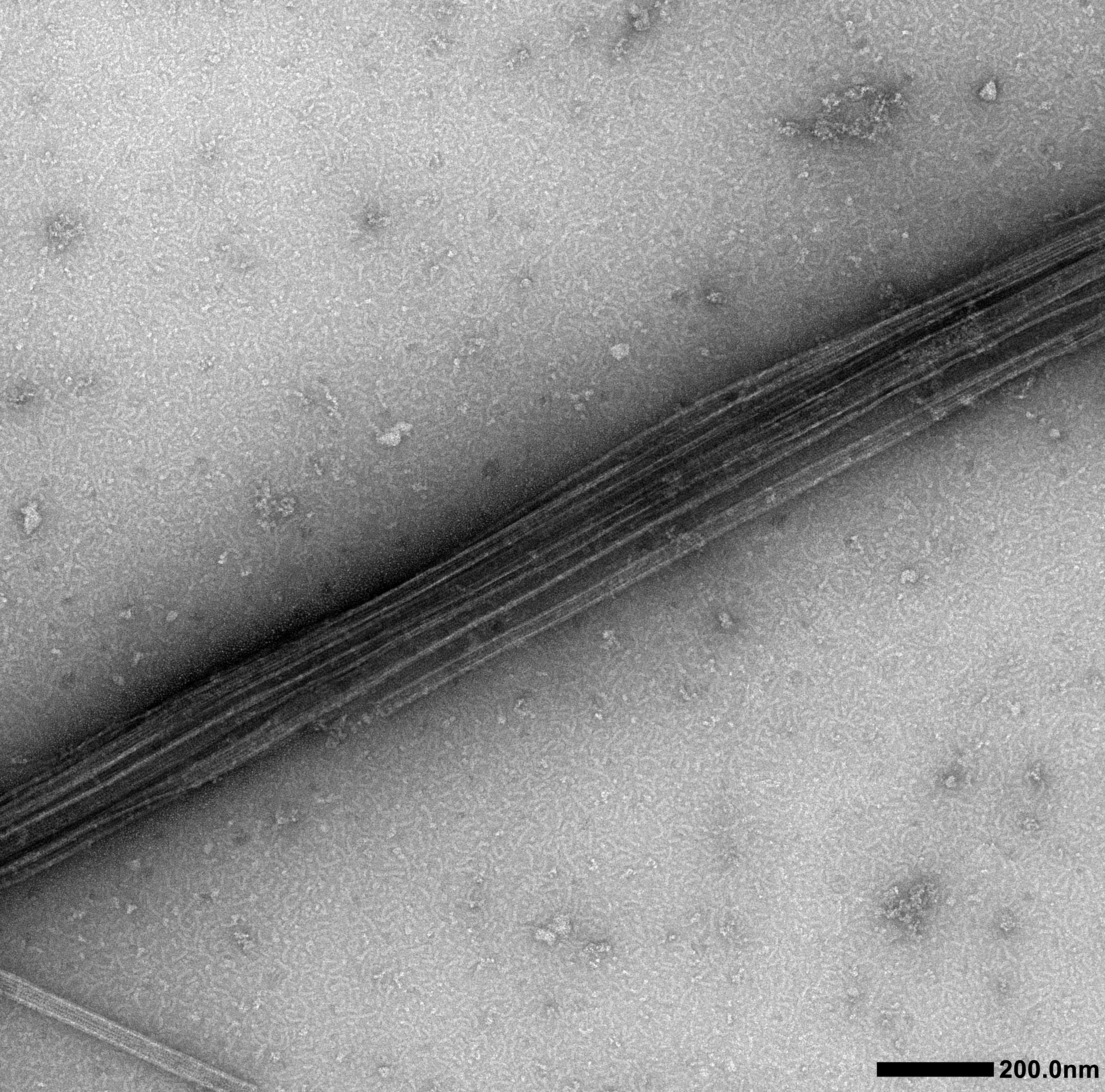

Supplement: Supplementary file 20 — Source data Fig. 6 [file 44318_2025_415_MOESM20_ESM.zip › Figure6/6C/Figure 6C.bmp]

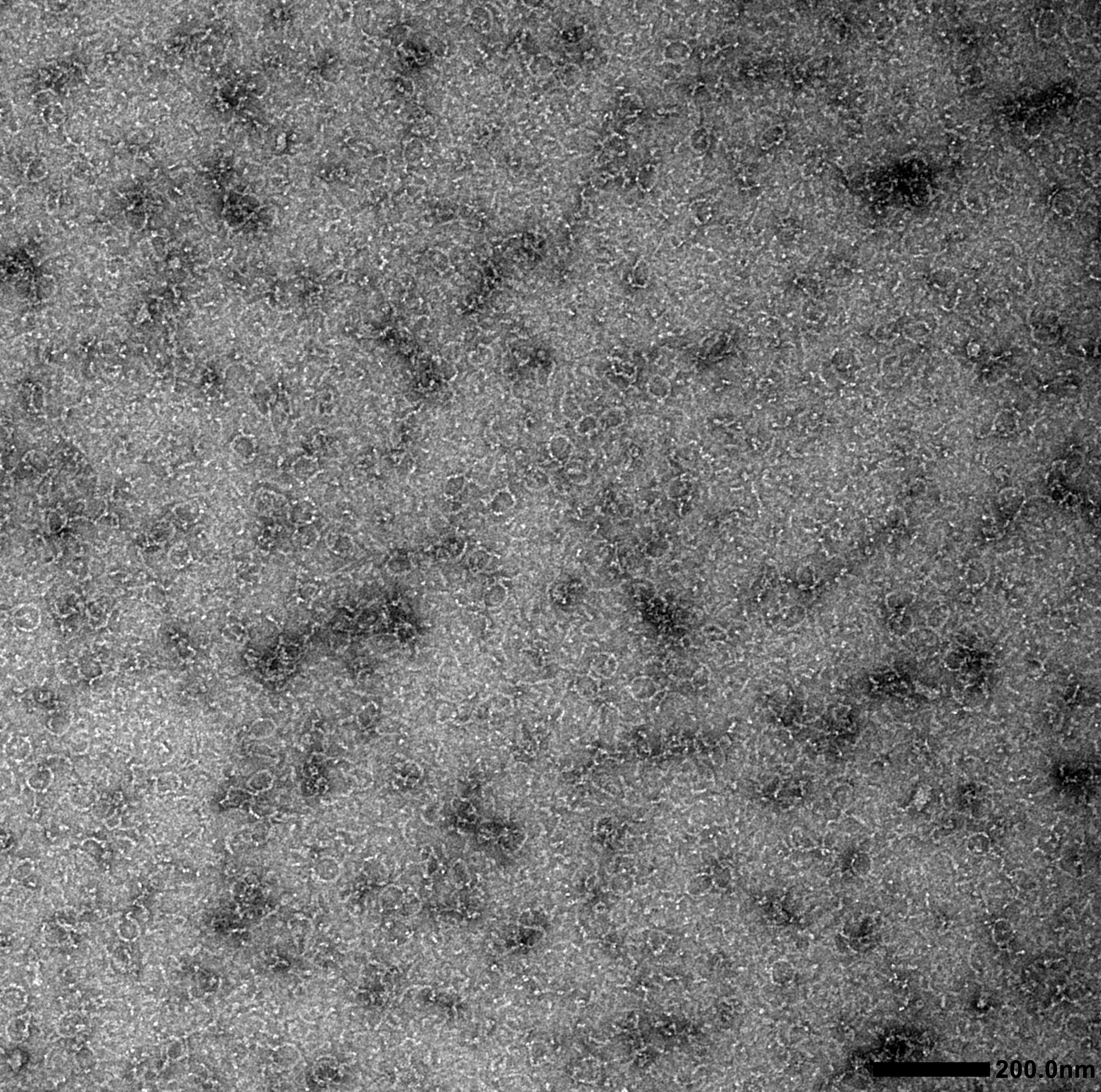

Supplement: Supplementary file 20 — Source data Fig. 6 [file 44318_2025_415_MOESM20_ESM.zip › Figure6/6E/Figure 6E.bmp]

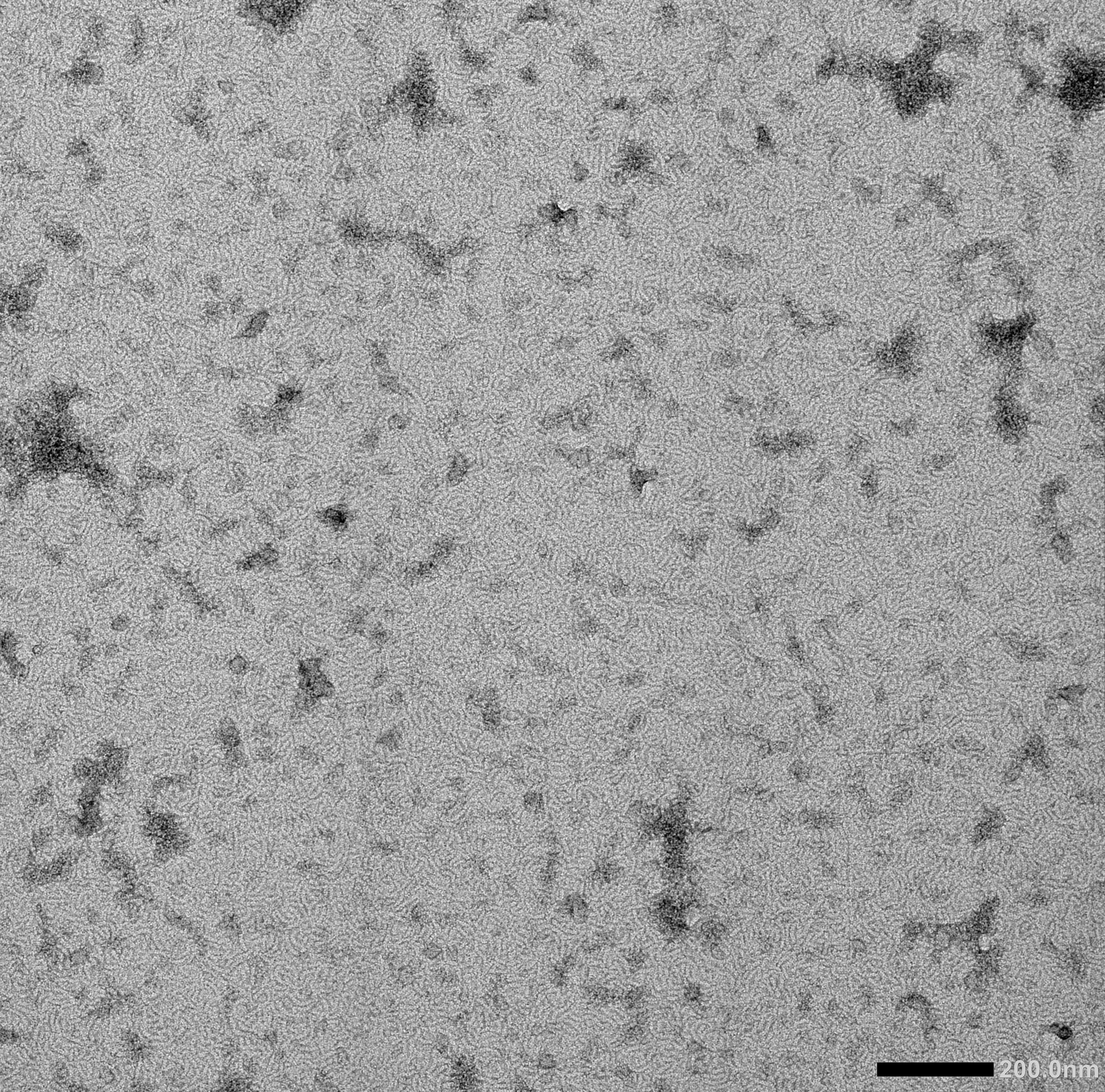

Supplement: Supplementary file 20 — Source data Fig. 6 [file 44318_2025_415_MOESM20_ESM.zip › Figure6/6F/Figure 6F.bmp]

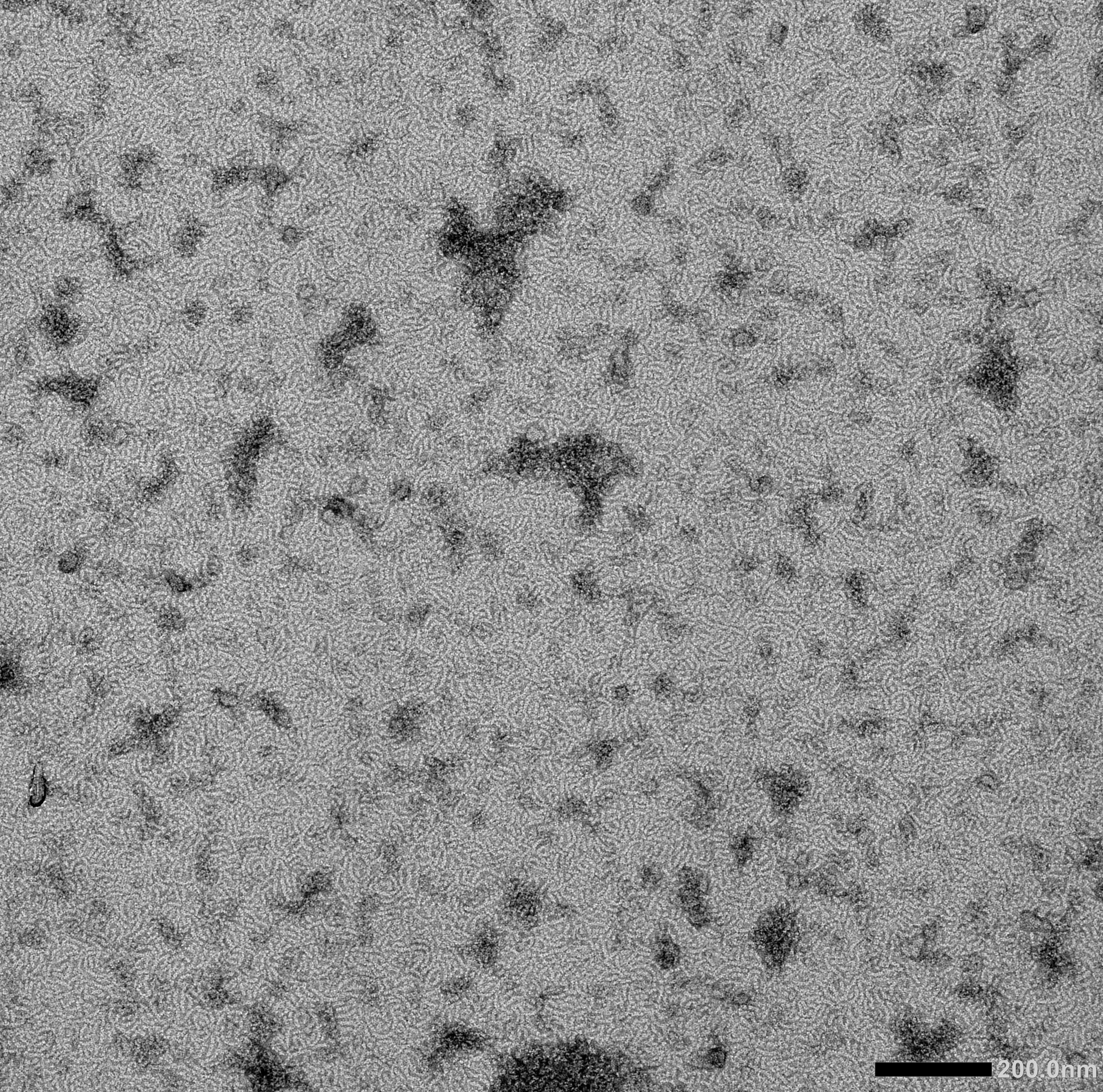

Supplement: Supplementary file 20 — Source data Fig. 6 [file 44318_2025_415_MOESM20_ESM.zip › Figure6/6G/Figure 6G.bmp]

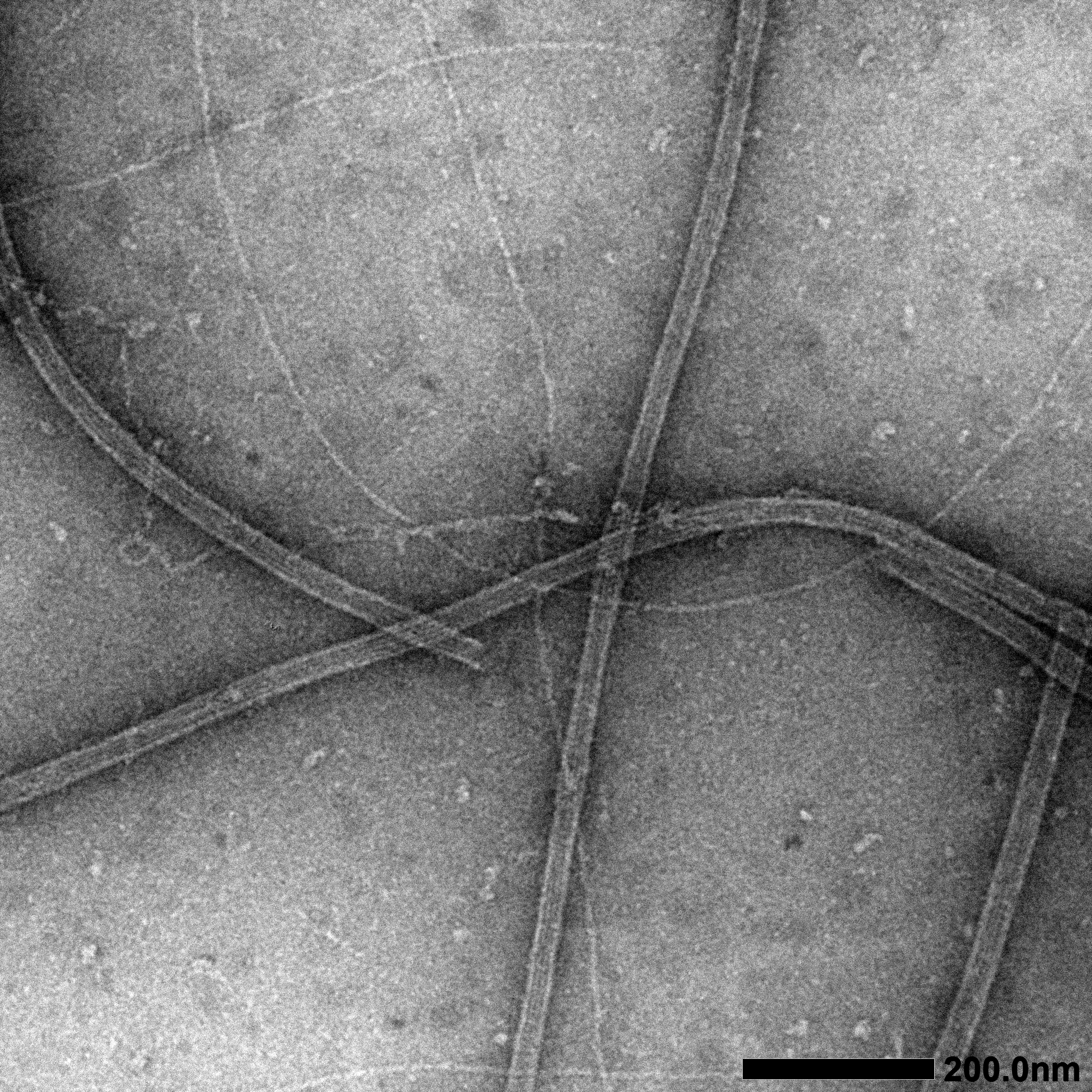

Supplement: Supplementary file 21 — Source data Fig. 7 [file 44318_2025_415_MOESM21_ESM.zip › Figure7/7A/Figure 7A.bmp]

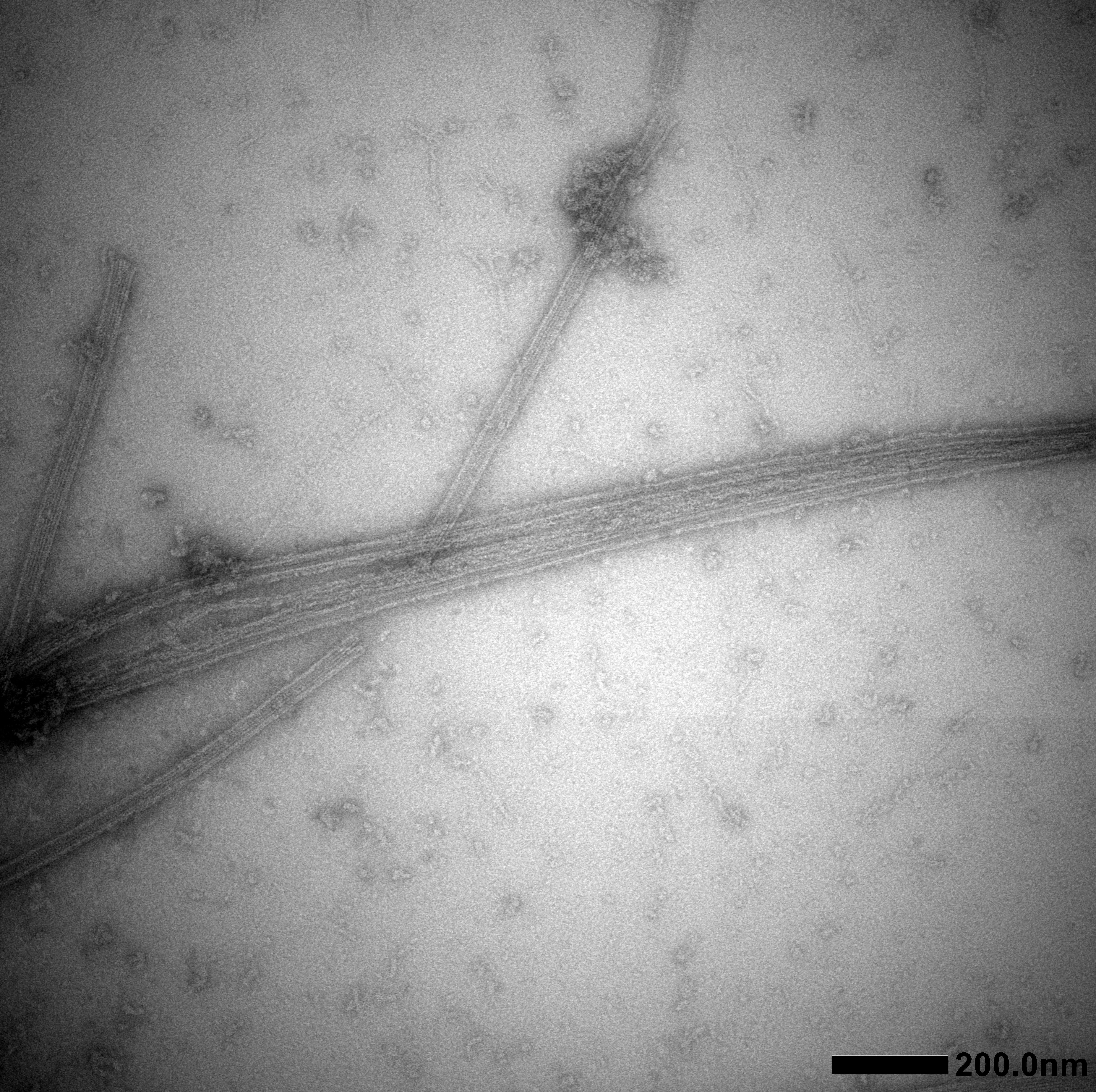

Supplement: Supplementary file 21 — Source data Fig. 7 [file 44318_2025_415_MOESM21_ESM.zip › Figure7/7B/Figure 7B.bmp]

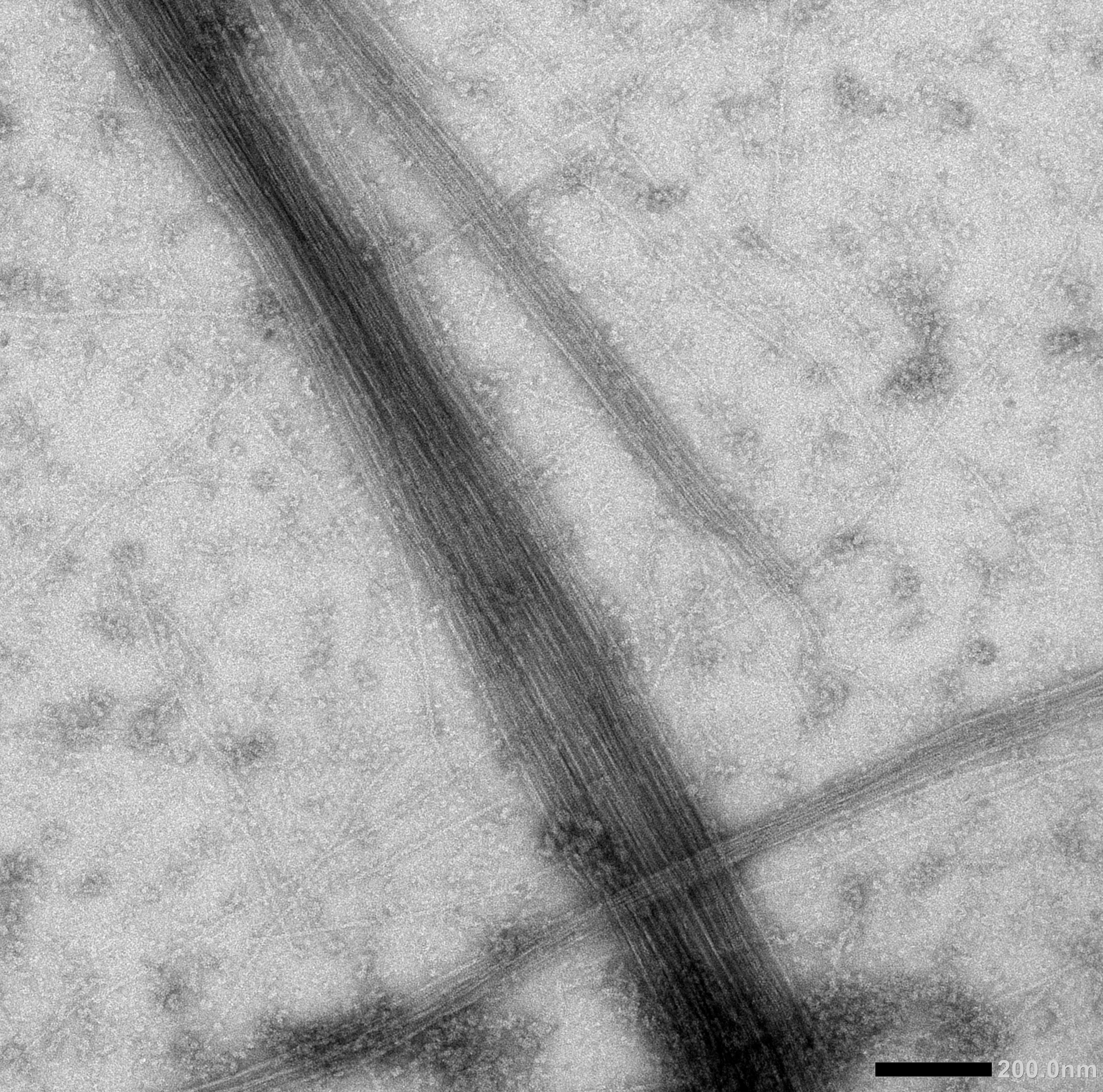

Supplement: Supplementary file 21 — Source data Fig. 7 [file 44318_2025_415_MOESM21_ESM.zip › Figure7/7C/Figure 7C.bmp]
