# Supplementary material for: Dimerization of GAS2 mediates crosslinking of microtubules and F-actin
Source: EMBO J. 2025 Apr 1;44(10):2997–3024. doi: 10.1038/s44318-025-00415-2 (PMC12084551; doi:10.1038/s44318-025-00415-2)
Supplement: Supplementary file 22 — Expanded View Figures [file 44318_2025_415_MOESM22_ESM.pdf]

Expanded View Figures

Figure EV1. Function of GAS2 protein in the cochlea, its purification, and assessment of its F-actin bundling activity.

(**A**) Domain structure of spectraplakins. (**B**) The schematic diagram illustrates the inner ear. Sound waves entering the outer ear vibrate the eardrum, with these vibrations amplified by the ossicles (malleus, incus, and stapes) and transferred to the oval window. The oval window transmits the vibrations to the cochlea, where fluid movement creates waves that cause the basilar membrane to vibrate (left panel). These vibrations are transduced into inner and outer hair cells (IHC, OHC) to generate an electrical signal supported by Pillar and Deiters' cells (middle panel). Pillar and Deiters' cells contain a dense cytoskeleton network; microtubules in tightly bundled square arrays spaced 120–150 Å apart and cross-linked with F-actin (left panel). This intricate structure is vital for sound wave oscillation by providing structural rigidity to the supporting cells. Three ncMTOCs (non-Centrosomal Microtubule-Organizing Centers) collaborate to coordinate control of microtubule positioning in the Pillar cells (middle panel). (**C**) SEC (Size Exclusion Chromatography) results for GAS2-FL, solid red line; GAS2ΔC, solid purple line; GAS2-GAR domain, solid green line; and GAS2-GARΔC, solid blue line with marker protein (black dot line). (**D**) The calibration formula for Superdex 75 HR 10/300 column according to the peak location of the marker proteins. (**E**) Calibration standard M.W. line for Superdex 75 HR 10/300 column according to peak location of marker proteins, shown as black dots and pink lines. The blue dot lines show the relation between the peak location and the molecular weight derived from the calibration standard line. (**F**, **G**) Representative Coomassie Blue-stained gels of co-sedimentation assays containing supernatant (S) and pellet (P) for purified GAS2-FL and GAS2-CH3 domain. (**H**, **I**) Low-speed co-sedimentation assay of purified full-length GAS2 (GAS2-FL) and C-terminal truncation GAS2ΔC. Various amounts of full-length GAS2 or GAS2ΔC were mixed with F-actin, incubated for 60 min at room temperature, and then centrifuged for 10 min at 9000 × *g*. Both supernatant (S) and pellet (P) were separated on SDS/PAGE and stained with CBB. All the raw SDS-PAGE gels are shown in Source data. Source data are available online for this figure.

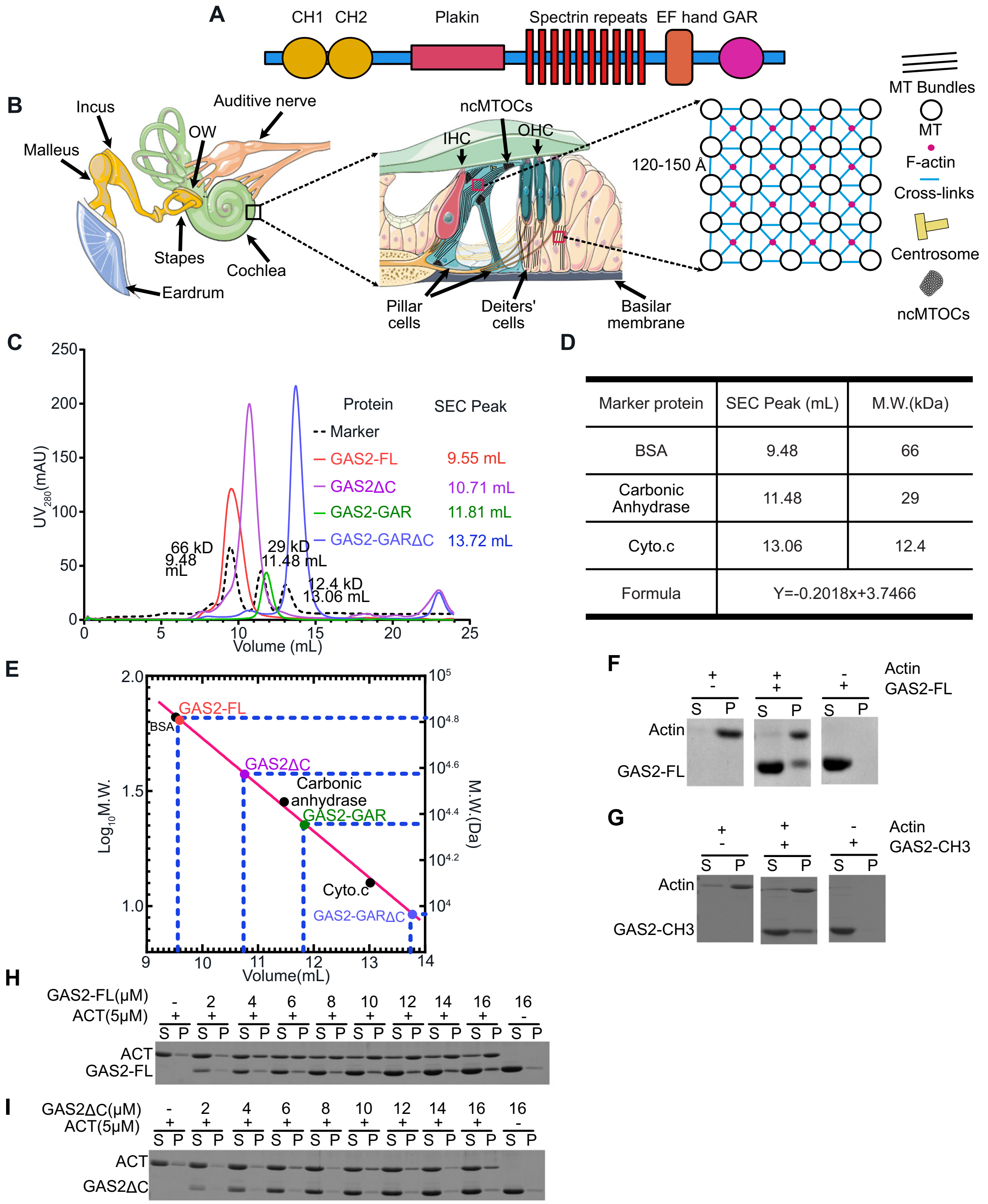

Marker protein	SEC Peak (mL)	M.W.(kDa)
BSA	9.48	66
Carbonic Anhydrase	11.48	29
Cyto.c	13.06	12.4
Formula	Y=-0.2018x+3.7466	

© The Author(s)

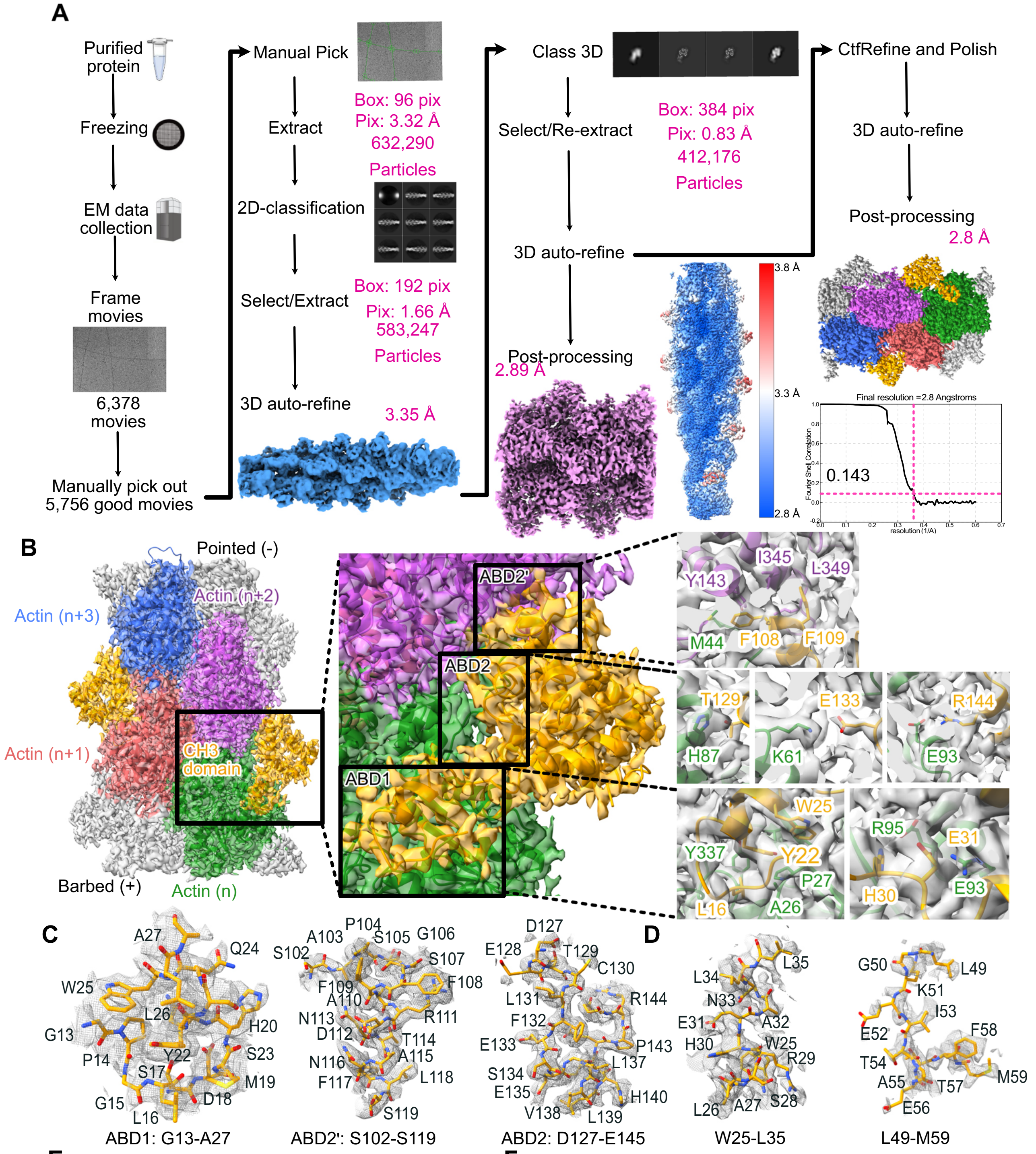

WT & Mutations	Mean-Kd(μM)
WT	5.26±0.36
L16D	9.13±0.86
Y22D	16.95±2.02
W25E	11.56±0.95
F108D	51.23±3.38
E133R	31.73±6.78
R144E	30.68±9.13
GAS2-CH3ΔN	24.11±4.38

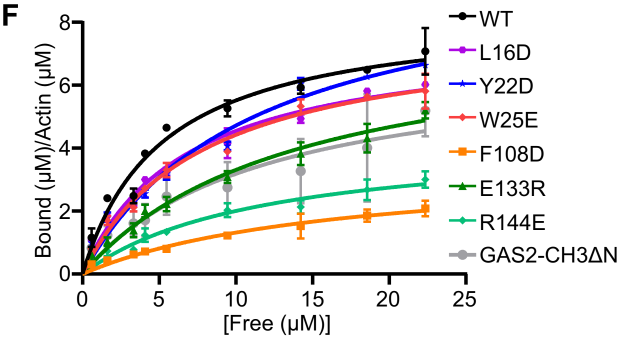

Figure EV2. Structural analysis of F-actin-GAS2-CH3 complex.

(**A**) GAS2-CH3-F-actin structure reconstruction workflow by RELION v4.0. (**B**) The atomic models for the GAS2-CH3 domain fitted into the 3D reconstruction density map and residual level information of key amino acids interacting with actin with density map same as (Fig. 2D). Actin subunits (marked as n series from bottom to top) are presented in green, Indian red, medium orchid, and royal blue. The GAS2-CH3 domain is presented in orange. (**C**) Model-map overlaps in good fitting examples for GAS2-CH3-F-actin complex, including ABD1: G13-A27; ABD2′: S102-S119; ABD2: D127-E145. (**D**) Model-map overlaps in bad-fitting examples for the GAS2-CH3-F-actin complex, including W25-L35 and L49-M59 peptides. Some density volumes are discontinuous. (**E**) The K_d value for WT and mutants of the GAS2-CH3 domain according to co-pellet assay. Each K_d value represents the mean ± SD. from three independent experiments. (**F**) The fitting curve for WT and mutants of the GAS2-CH3 domain for co-pellet assay. One site-specific binding method was ustoffiting the curve. Each data point represents the mean ± SD from three independent experiments. All the raw SDS-PAGE gels are shown in Source data.

© The Author(s)

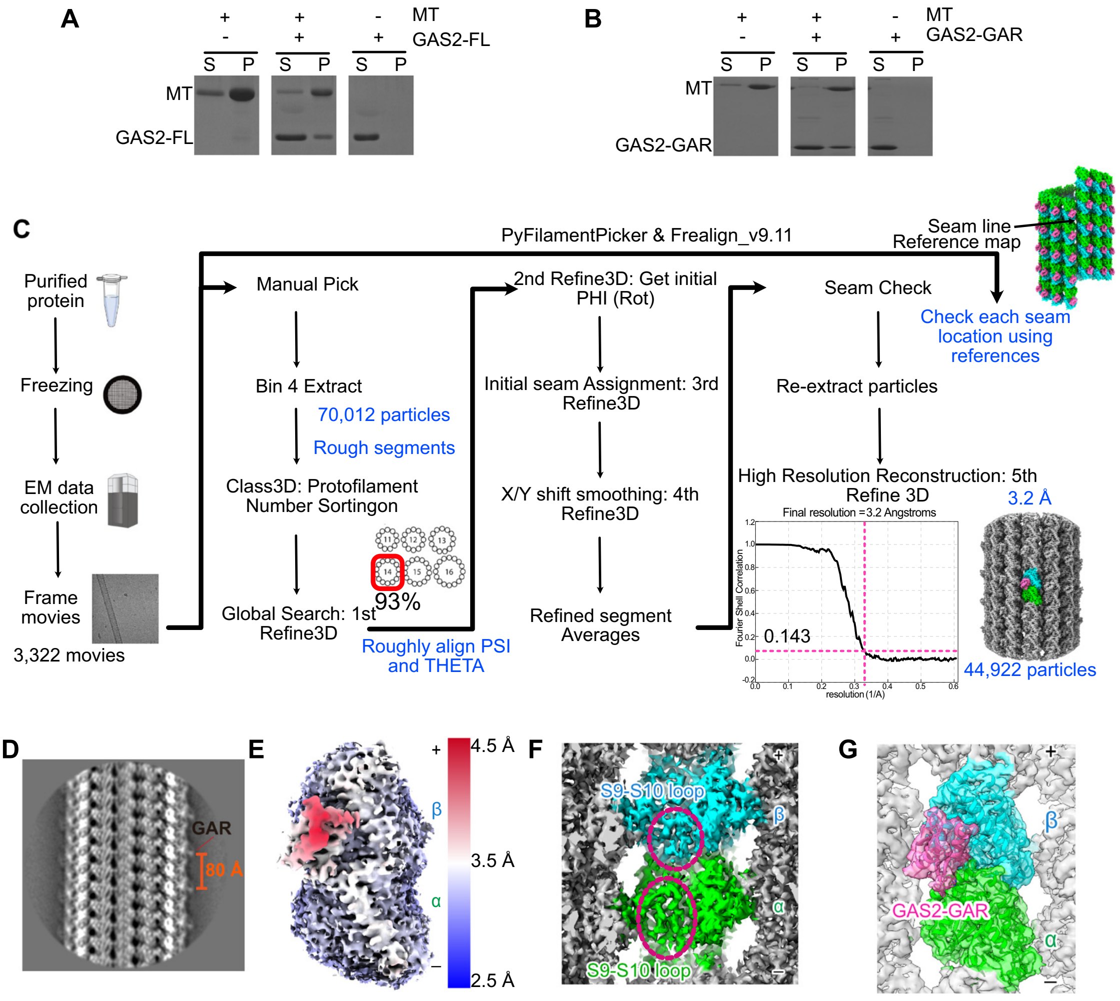

H

WT & Mutations	Mean-Kd(μM)
WT	0.69±0.09
R242E	5.00±0.91
R252E	46.87±9.23
G255A	2.69±0.34
G255E	9.60±2.00
W257E	26.85±4.76
K266E	10.58±1.65

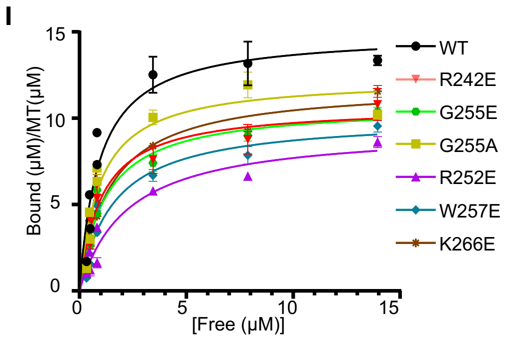

Figure EV3. Structural analysis of MT-GAS2-GAR complex.

(**A**, **B**) Representative Coomassie-stained MT co-sedimentation assay gels containing supernatant (S) and pellet (P) for purified GAS2-FL and GAS2-GAR domain. (**C**) GAS2-GAR-MT structure rebuilding workflow by MiRP. (**D**) The 2D class average for a GAS2-GAR decorated on a microtubule. GAS2-GAR domains are regularly spaced at 8 nm. (**E**) The local resolution estimation of the asymmetric units by RELION v3.1.3 for MiRP method reconstructed density map. Blue represents 2.5 Å, white represents 3.5 Å, red represents 4.5 Å. (**F**) Zoomed-in view of the cryo-EM reconstruction from the lumen for MiRP method reconstructed GAS2-GAR-MT density map. Red dashed circles highlight the density regions corresponding to the S9-S10 loop, which has different lengths in α- and β-tubulin (α-tubulin has a longer S9-10 loop), aiding in subunit assignment. (**G**) Outside top view of atomic models for GAS2-GAR domain fitted in the 3D reconstruction density map by MiRP (α-tubulin in green, β-tubulin in cyan, GAS2-GAR domain in hot-pink). (**H**) The K_d value for WT and mutants of the GAS2-GAR domain according to co-pellet assay. Each K_d value represents the mean ± SD from three independent experiments. (**I**) The fitting curve for WT and mutants of the GAS2-GAR domain for co-pellet assay. One site-specific binding method was used to fit the curve. Each data point represents the mean ± SD from three independent experiments. All the raw SDS-PAGE gels are shown in Source data. Source data are available online for this figure.

© The Author(s)

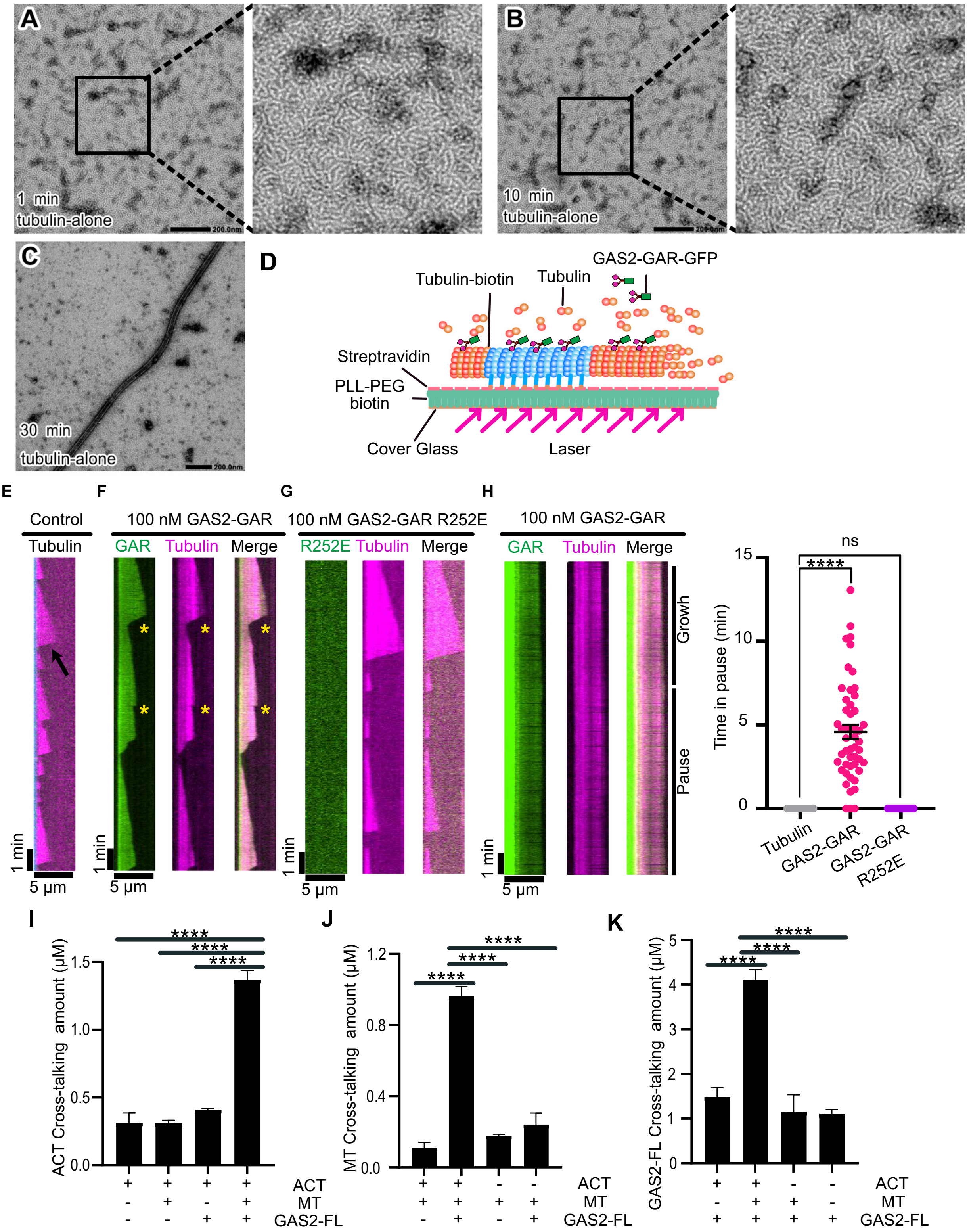
A
1 min
tubulin-alone
B
10 min
tubulin-alone
C
30 min
tubulin-alone
D
GAS2-GAR-GFP
Tubulin-biotin
Tubulin
Streptravidin
PLL-PEG
biotin
Cover Glass
Laser
E
F
G
H
Control
Tubulin
100 nM GAS2-GAR
GAR
Tubulin
Merge
100 nM GAS2-GAR R252E
R252E
Tubulin
Merge
100 nM GAS2-GAR
GAR
Tubulin
Merge
Growth
Pause
1 min
5 μm
ns
****
Time in pause (min)
Tubulin
GAS2-GAR
GAS2-GAR R252E
I
J
K
ACT Cross-talking amount (μM)
MT Cross-talking amount (μM)
GAS2-FL Cross-talking amount (μM)
ACT
MT
GAS2-FL

 © The Author(s)

Figure EV4. The GAS2 protein facilitates microtubule nucleation and stabilization while also crosslinking F-actin and microtubules.

(**A–C**) Representative negative stain EM micrographs of 3 μM tubulin-alone polymerization at different time points. (**D**) Schematic of TIRF microscopy-based MT stabilization assay. The biotin-labeled microtubule seed (cyan) was tethered to the cover glass by streptavidin and PLL-PEG biotin. The free tubulin dimers (shown in red and orange color) polymerize or depolymerize at the end of the microtubule seed. (**E**) A representative kymograph of microtubules (magenta) growing from GMPCPP seeds (cyan) in tubulin-alone. The black arrow shows one of the catastrophe events. (**F**) Representative kymographs of one microtubule (magenta) growing in the presence of GAS2-GAR-GFP (green). Yellow stars represent the rescue event. From left to right, the panels show the GAS2-GAR-GFP, tubulin (magenta), and the merged kymographs. (**G**) Representative kymographs of one microtubule (magenta) growing in the presence of GAS2-GAR-R252E-GFP (green). From left to right, the panels show the GAS2-GAR-R252E-GFP, tubulin (magenta), and the merged kymographs. (**H**) The left panel shows the representative kymograph of the pause state of microtubule dynamics in the presence of GAS2-GAR-GFP. "Growth" represents MT polymerization. "Pause" represents the MT pause state. The right panel shows the average pause time per microtubule. The black bars show mean ± SEM. For control, bare-MTs ($N = 19$) were analyzed. For GAS2-GAR-GFP, $N = 50$ MTs were analyzed. For GAS2-GAR-R252E-GFP, $N = 21$ MTs were analyzed. One-way ANOVA with Dunnett's multiple comparisons test has been used. **** p-value < 0.0001. p values were calculated relative to the tubulin-alone condition. p-value from left to right for pause events:4.6E-11, 0.99. For all kymographs, horizontal scale bar = 5 μm; vertical scale bar = 1 min. (**I–K**) Histogram of pellet quantitative analysis for F-actin, MT, and GAS2-FL in (Fig. 7E). Each value represents the mean ± SD. from three independent experiments. One-way ANOVA with Dunnett's multiple comparisons test has been used. ****p-value < 0.0001. p-value in (**I**) from left to right: 2.4E−8, 2.3E−8, 5.0E−8; p-value in (**J**) from left to right: 3.8E−8, 7.3E−8, 1.4E−6; p-value in (**K**) from left to right: 4.1E−6, 1.6E−6, 4.1E−6.

© The Author(s)

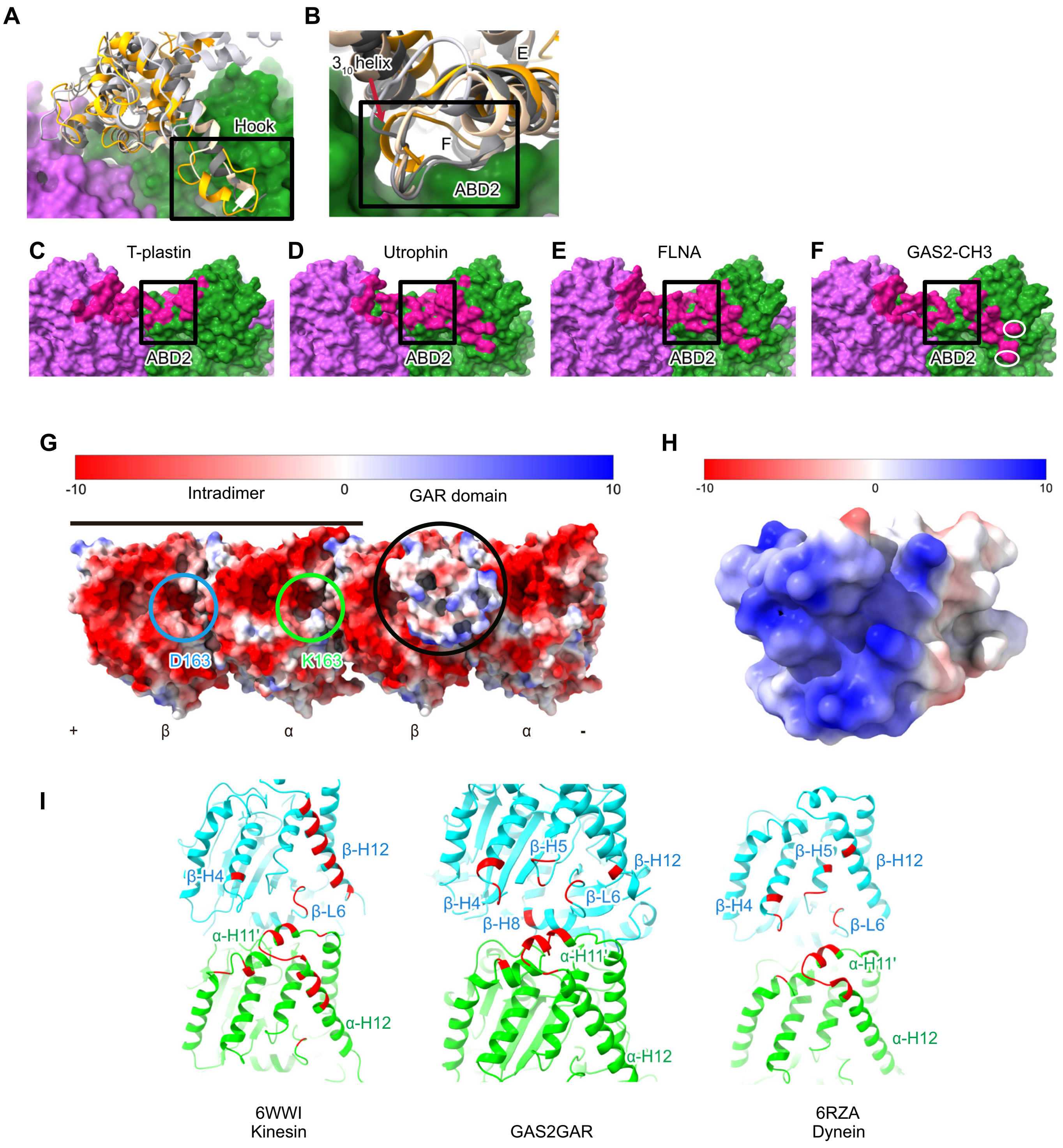

Figure EV5. Structural comparison of CH domains and electrostatic interactions analysis of GAS2-GAR with microtubules.

(**A**) CH domain N-terminal structure alignment, GAS2-CH3 (orange) interacts with actin by a longer N-terminal compared with T-plastin (silver, PDB:7R94, EMDB: EMD-24323), utrophin (gray, EMDB: EMD-30085, PDB: 6M5G) and FLNA (wheat, EMDB: EMD-7831, PDB:6D8C). (**B**) GAS2-CH3 domain rigid 3_{10} α-helix structure compares with the loops of T-plastin, utrophin, and FLNA by the same color scheme and structure data bank source as in (**A**). (**C–F**) Footprint method to inspect potential contacts between actin and the binding partner as defined within 5-Å distance by ChimeraX with the same structure data bank source as in (**A**). (**G**) Overall analysis of the electrostatic potential of the contact surfaces showed complementary charges between the GAS2-GAR domain and the MT. The black circle shows the GAS2-GAR domain well bound to charge complementary concave of the intra-tubulin dimer. The cyan box shows negatively charged amino acid D163 in β-tubulin. The lime box shows the positively charged amino acid D163 in α-tubulin. (**H**) The bottom of the GAR domain is full of positively charged amino acids. (**I**) Footprint method to inspect potential contacts between tubulin and the binding partner as defined within 5-Å distance by ChimeraX, Kinesin PDB:6WWI, and Dynein PDB: 6RZA were used.

 © The Author(s)
